# Standardized patient profile review using large language models for case adjudication in observational research

**DOI:** 10.1038/s41746-025-01433-4

**Published:** 2025-01-09

**Authors:** Martijn J. Schuemie, Anna Ostropolets, Aleh Zhuk, Uladzislau Korsik, Seung In Seo, Marc A. Suchard, George Hripcsak, Patrick B. Ryan

**Affiliations:** 1 Observational Health Data Science and Informatics, New York, NY USA; 2 Global Epidemiology Organization, Johnson & Johnson, Titusville, NJ USA; 3 Department of Biostatistics, UCLA, Los Angeles, CA USA; 4 Department of Biomedical Informatics, Columbia University Irving Medical Center, New York, NY USA; 5 Odysseus Data Services, Cambridge, MA USA; 6 Division of Gastroenterology, Department of Internal Medicine, Kangdong Sacred Heart Hospital, Hallym University College of Medicine, Seoul, Republic of Korea

**Keywords:** Epidemiology, Computer science

## Abstract

Using administrative claims and electronic health records for observational studies is common but challenging due to data limitations. Researchers rely on phenotype algorithms, requiring labor-intensive chart reviews for validation. This study investigates whether case adjudication using the previously introduced Knowledge-Enhanced Electronic Profile Review (KEEPER) system with large language models (LLMs) is feasible and could serve as a viable alternative to manual chart review. The task involves adjudicating cases identified by a phenotype algorithm, with KEEPER extracting predefined findings such as symptoms, comorbidities, and treatments from structured data. LLMs then evaluate KEEPER outputs to determine whether a patient truly qualifies as a case. We tested four LLMs including GPT-4, hosted locally to ensure privacy. Using zero-shot prompting and iterative prompt optimization, we found LLM performance, across ten diseases, varied by prompt and model, with sensitivities from 78 to 98% and specificities from 48 to 98%, indicating promise for automating phenotype evaluation.

## Introduction

In the realm of healthcare research, the reuse of data, such as administrative claims and electronic health records (EHRs), for observational studies has become commonplace. However, these datasets, not originally collected for research purposes, often lack precise information required to address specific research questions. Researchers often must infer important variables such as exposures and outcomes from available markers such as diagnosis codes and laboratory tests. To extract health outcomes of interest, guidelines recommend first crafting conceptual case definitions and then deriving operational definitions, containing specific codes such as ICD-10 and LOINC to look for, and the logic to combine them^1^. These operational definitions are often referred to as phenotype algorithms.

The validity of observational research hinges on the accuracy of these algorithms, a critical aspect addressed through outcome (and similarly exposure) validation. Typically, this involves labor-intensive chart review, scrutinizing clinical details to ensure the operational definition accurately represents the conceptual case definition. Yet, chart review is time-consuming, subjective, and lacks portability between datasets. FDA guidelines^1^ advocate for comprehensive chart review of all potential cases, but the practicality of this approach is limited. In practice, often only a small sample of identified cases is reviewed, allowing only the positive predictive value (PPV) to be computed, which is insufficient to fully determine potential bias from outcome misclassifications^2^. At the very least, guidelines recommend reviewing a sample of both identified cases and non-cases, allowing for quantitative bias analysis, which is rarely feasible for rare outcomes.

**Fig. 1:**
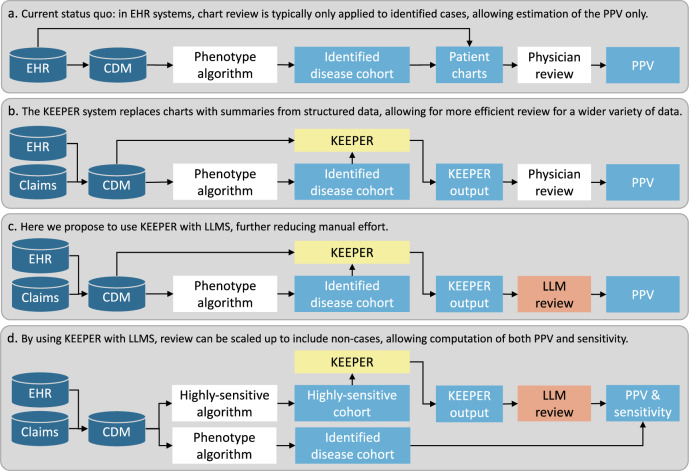
Overview of the various existing and proposed workflows. EHR = Electronic Health Records, CDM = Common Data Model, PPV = Positive Predictive Value. **a** Current status quo: in EHR systems, chart review is typically only applied to identified cases, allowing estimation of the PPV only. **b** The KEEPER system replaces charts with summaries from structured data, allowing for more efficient review for a wider variety of data. **c** Here we propose to use KEEPER with LLMS, further reducing manual effort. **d** By using KEEPER with LLMS, review can be scaled up to include non-cases, allowing computation of both PPV and sensitivity.

In response to these challenges, our previous work introduced the Knowledge-Enhanced Electronic Profile Review (KEEPER) system, as illustrated in Fig. 1b and described in the Methods section. KEEPER is a phenotype evaluation tool that extracts patient’s structured data elements relevant to a phenotype and presents them in a standardized fashion following clinical reasoning principles, allowing chart reviewers to adjudicate cases more accurately and efficiently^3^. KEEPER demonstrated high agreement with manual chart review, while reducing time needed to review and increasing inter-annotator agreement, alleviating some challenges associated with traditional chart review methods. Another benefit of KEEPER is that it utilized the Observational Medical Outcomes Partnership (OMOP) Common Data Model (CDM), thereby allowing review for data sources that do not readily provide charts, such as administrative claims.

Building upon this foundation, we extend the scalability and cost-effectiveness of KEEPER by incorporating large-language models (LLMs), to adjudicate cases based on KEEPER’s output. LLMs, recognized for their potential applications in the medical domain^4–6^, offer a promising avenue for automating the adjudication process. Given privacy constraints, we evaluate in-house hosted LLMs—specifically, the commercially available GPT-3.5 Turbo and GPT-4, and the freely available Llama-2 and Sheep-Duck-Llama-2.

To illustration our approach: one of the diseases evaluated in our study is osteoporosis, defined conceptually (per Supplementary Table 3) as “a skeletal disorder characterized by decreased bone density and strength, leading to fragile bones and an increased risk of fractures.” Our phenotype algorithm identifies cases by the first recorded diagnosis code that maps to the standard concept of Osteoporosis or any of its descendants within the CDM, facilitating cross-system compatibility^7^. For example, this standard concept encompasses 223 ICD-10 codes, including M81.9 (Osteoporosis, unspecified) and M81.0 (Postmenopausal osteoporosis). Running this algorithm identifies patients with an index date marking the point at which they are believed to have osteoporosis. To assess the algorithm’s operating characteristics in a specific data source, we could use either manual chart review or the KEEPER system. We defined sets of concepts for KEEPER to extract (see Supplementary Note 2) from certain time windows (Supplementary Table 1). These include related conditions (e.g., “Malignant neoplastic disease” within 90 days of index), symptoms (e.g., “Joint pain of pelvic region” within 30 days before index), comorbidities (e.g., “Fracture of bone” at any point prior), and relevant treatments (e.g., “Zoledronic acid” prescribed after diagnosis). Prior research suggests that KEEPER’s output provides enough information for human reviewers to confirm or refute osteoporosis case status. Here, we investigate how accurately a large language model (LLM) can make this determination based on KEEPER’s output.

This manuscript investigates whether case adjudication using KEEPER and LLMs is feasible and could serve as a viable alternative to manual chart review. We outline the prompt engineering guided by a development set specifically created for this purpose. We then assess performance of the optimal prompting strategy on three test sets, comparing results to gold standards derived from human annotation. Additionally, we demonstrate how the combination of KEEPER and LLMs can be used to create a large silver standard without human intervention, allowing estimation of both positive predictive value and sensitivity of phenotype algorithms. The Methods section describes the KEEPER system and outlines the creation of the development and test sets.

## Results

### Prompt engineering

Using a dedicated human-annotated development set to guide our prompt engineering, we initiate our approach with a basic system prompt, instructing the LLM to deliver a binary ‘yes’ or ‘no’ response to the question of whether the patient had the disease of interest. Following the concept of ‘chain-of-thought’^8^, we progressively enhanced the prompt. We introduced text prompting the LLM to initially present evidence both in favor and against the specified disease. Additionally, we requested the LLM to generate a clinical narrative aligning with the provided data. Observing a tendency for the LLM to heavily weigh diagnoses, leading to frequent false positive classifications, we adjusted the system prompt to emphasize that a single diagnosis does not conclusively indicate the case as true. Subsequently, we identified inconsistency in how the LLM handled uncertainty, occasionally responding ‘yes’ even when another diagnosis was more likely or ‘no’ in the presence of unreasonable doubt. To address this, explicit instructions on handling uncertainty were incorporated into the prompt. To enhance performance, we further introduced two examples through few-shot prompting. The prompting strategy demonstrating the highest area under the receiver operator curve (AUC) on the development set, shown in Table 1, was selected as optimal.

**Table 1:** Performance of various prompting strategies and LLMs on the development set

Prompt (using GPT 4)	Sensitivity	Specificity	AUC
Yes/no	98.5%	3.2%	0.51
+ discuss evidence	98.0%	7.3%	0.53
+ write narrative	97.5%	19.4%	0.58
+ diagnosis insufficient reminder	81.9%	83.1%	0.82
+ uncertainty instructions	86.8%	83.9%	0.85
+ provide examples	82.8%	73.4%	0.78
**LLM (using all but last prompt)**			
GPT 4	86.8%	83.9%	0.85
GPT 3.5 Turbo	82.8%	58.9%	0.71
Llama-2-70b-chat-hf	99.0%	12.9%	0.56
Sheep-Duck-Llama-2-70b-v1.1	90.2%	62.1%	0.76

*AUC* Area under the receiver operator curve.

**Fig. 2:**
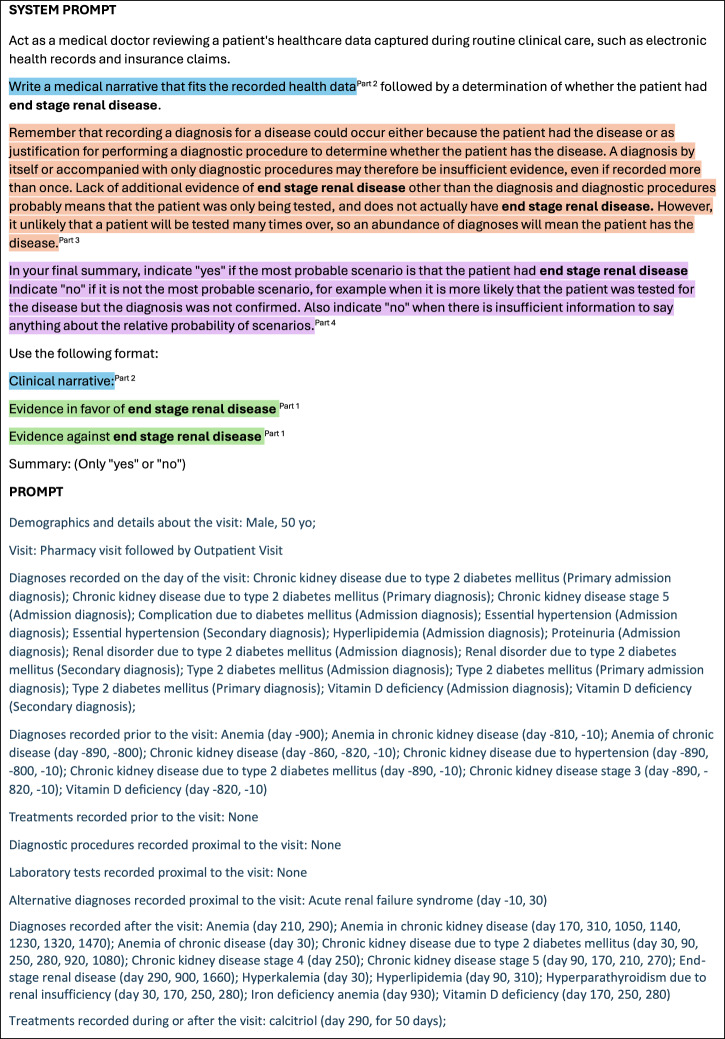
Final system prompt and example main prompt. The finalized system prompt with an example main prompt. Part 1 of the system prompt instructs the LLM to discuss evidence for and against the disease. Part 2 prompts the system to generate a clinical narrative. Part 3 reinforces that a diagnosis alone is insufficient evidence, and Part 4 provides guidance on handling uncertainty. To safeguard patient privacy, this figure shows perturbed patient data. The LLMs were provided with the unperturbed real data.

The finalized system prompt, outlined in Fig. 2, encompasses four parts. Part 1 instructs the LLM to discuss evidence for and against the disease. Part 2 prompts the system to generate a clinical narrative. Part 3 reinforces that a diagnosis alone is insufficient evidence, and Part 4 provides guidance on handling uncertainty. The Supplementary Note 6 contains the response of GPT-4 to the prompt in Fig. 1.

After determining the optimal prompt strategy using GPT-4, we evaluated this strategy using Llama-2, which exhibited poor performance. Consequently, we opted for Sheep-Duck-Llama-2 (SDL2), as indicated in Table 1. For completeness, we also evaluated GPT-3.5 Turbo. Running GPT-4 on the development set took approximately 102 min, GPT-3.5 took 48 min. Running Llama-2 took 50 h, and running SDL2 on the development set took 66 h. However, these numbers are subject to change, as LLMs are continuously improving in speed, and both Llama-2 and SDL2 can now be hosted in the cloud as well.

### Performance on test sets

**Fig. 3:**
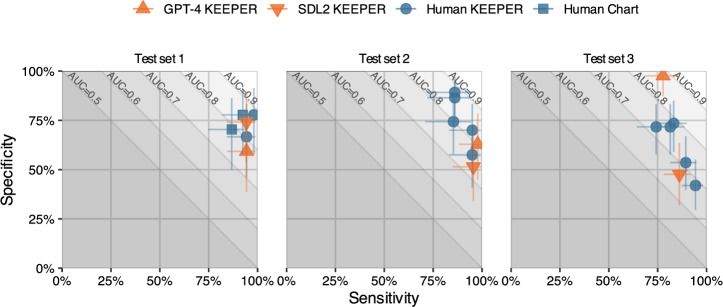
Sensitivity and specificity of reviewers for the three test sets. Points indicate sensitivity and specificity of each human or LLM reviewer against the gold standard. Error bars indicate 95% confidence intervals. For test set 1, the gold standard was created by external reviewers. For test set 2 and 3, the gold standard was the majority vote of human reviewers using a leave-one-out approach. Slanted lines denote iso-AUC contours, spaced 0.1 apart.

Fig. 3 illustrates the performance of human reviewers compared to the optimal prompt strategies applied to GPT-4 and SDL2. When evaluated against the gold standard for test set 1, LLMs demonstrate similar levels of sensitivity and specificity to those of human reviewers. In test set 2, LLMs show higher sensitivity, though their specificity falls at the lower range of human performance. For test set 3, GPT-4 achieves near-perfect specificity, albeit with sensitivity at the lower range seen among human reviewers, while SDL2 displays higher sensitivity but substantially lower specificity. We observe that LLMs generally achieve similar AUC values to human reviewers, although with some variability in sensitivity–specificity trade-offs.

For test sets 1 and 2, performance did not vary much per disease (see Supplementary Notes 7 and 8). For test set 3 performance did vary greatly per disease, both for humans and LLMs, with lower sensitivity and specificity for acute bronchitis and viral hepatitis A (see Supplementary Note 9).

### Performance on the highly sensitive set

GPT-4 required 92 h to annotate a highly sensitive set of 25,000 potential rheumatoid arthritis (RA) cases, requiring approximately 20 million tokens at the cost of about $900 at the time of execution (Februari 2024). From this set, only 360 patients (1.4%) were classified as true cases. Using these 25,000 annotated patient records as a silver standard, we evaluated the OHDSI RA phenotype algorithm, revealing a PPV of 56.5% (95% confidence interval: 52.4 – 60.5%) and a sensitivity of 93.0% (95% CI: 89.9–95.5%).

## Discussion

In our findings, zero-shot prompting, with slight modifications to the system prompt, demonstrated reasonable performance in case adjudication using the KEEPER system, with comparable agreement with the gold standard as human reviewers. The performance of LLMs was notably influenced by the choice of prompt and the specific LLM selected. Disease-dependent variations in adjudicator performance, even among human reviewers, highlight the inherent uncertainty in the task of case adjudication, possibly reflecting the inherent uncertainty in disease diagnosis itself. For example, sensitivity and specificity for viral hepatitis A tended to be low, most likely because the data lacked results of laboratory tests, which made it hard to interpret orders of panels for different hepatitis.

Within these inherent limits, LLMs can contribute to evaluating the veracity of a phenotype algorithm to assess the evidence supporting a case’s authenticity. Various use cases emerge depending on how one perceives value of LLM outputs—from using LLMs as a co-pilot for human assessment to fully automating adjudication for estimating operating characteristics of the phenotype algorithm in each database. The combined use of KEEPER with LLMs facilitates the quick adjudication of large case volumes, enabling the computation of both PPV and sensitivity for numerous phenotypes and data sources. In our example, we observed a PPV of 56.6% and sensitivity of 93.0% for a specific RA phenotype algorithm in a specific database. Armed with these operating characteristics, a researcher could decide to not use this phenotype algorithm, or take them into account in some form of quantitative bias analysis^2^.

We note that, despite having adjudicated 25,000 cases, the confidence intervals around PPV and sensitivity in our demonstration are still wide, arguing for even larger samples of cases to be adjudicated. This would certainly be prohibitively expensive with a manual approach. For context, a typical price charged for manual chart review is US$100 per case, or US$2.5 million for 25,000 cases. The cost for the LLM at the time for the same task was US$900, and prices for LLM usage have significantly dropped since then. For privacy reasons, patient-level data should be kept within an organizational firewall. Fortunately, many LLMs such as SDL2 are freely available for on-premises hosting, and many commercial models offer within-firewall cloud-based solutions.

Combining LLMs with KEEPER enables our approach to be applied to any data within the CDM, including both administrative claims and EHRs. Our results on Test set 1 indicate that the performance of this combined method is comparable to human reviewers using KEEPER, and even to human reviewers with access to full patient charts. Although LLMs could theoretically be applied to entire charts, this is currently impractical with the models we evaluated, as the charts exceed the context window limit (e.g., 4000 tokens for Llama-2). Newer models introduced since the completion of this work boast larger context windows, typically up to 125,000 tokens, but this would still be insufficient to include the full charts of a patient. While access to full charts might improve performance, the original KEEPER experiment suggests that more information does not necessarily enhance accuracy.

The work described here represents substantial effort in manual case adjudication; The development set required 358 reviews, test set 1 required 4 reviewers x 4 diseases x 20 cases = 320 case reviews, test sets 2 required 5 × 4 x 20 = 400 reviews, and test set 3 required 5 × 6 x 25 = 750 reviews. The total number of manual reviews is therefore 1828. Despite all this work, we were only able to evaluate our approach on 10 different diseases, with only a handful of cases per disease, limiting our ability to generalize from our findings.

While our current approach uses zero-shot and one attempt at few-shot prompting, possibly better performance could be achieved using automated prompt optimization^9^ or fine-tuning of the LLMs for the task at hand. However, this would require a large training set that would itself be infeasible due to cost and time constraints.

While the adoption of LLMs in clinical care remains debated, our application in enhancing evidence reliability from observational data appears promising and low risk.

## Methods

### The KEEPER system

KEEPER is fully described previously^3^. In brief, the design of KEEPER is guided by three fundamental principles: adherence to clinical reasoning, standardization, and dimensionality reduction.

1. Adherence to Clinical Reasoning:
  KEEPER is constructed to emulate the diagnostic clinical reasoning process when applied to patient data by organizing structured data based on clinical presentation, disease history, preliminary diagnosis, diagnostic procedures, differential diagnoses, treatment, follow-up care, and complications.
2. Standardization:
  To ensure scalability and applicability across diverse data sources, data extraction is based on the OMOP CDM^10^. KEEPER’s outputs are standardized across diseases.
3. Dimensionality Reduction:
  KEEPER focuses on efficiency by extracting only clinically relevant information for a specific phenotype, reducing data volume and expediting review.

In practice, KEEPER uses structured data in a common format to extract pre-specified elements relevant to the disease during standard time-windows aligned with typical clinical reasoning steps. Configuring KEEPER for a specific outcome requires specifying the elements – captured as sets of standard concepts in the OHDSI Standardized Vocabularies^7^ -- to extract per KEEPER category. For example, for acute appendicitis the ‘Symptoms’ category would specify concepts such as ‘nausea’, ‘vomiting’, and ‘epigastric pain’, and KEEPER would report if these concepts occurred in the 30 days prior to the case index date. The Supplementary Table 1 contains a full list of all KEEPER categories and corresponding time windows. KEEPER is applied to a cohort of patients, for example those identified by a phenotype algorithm.

### Large language models

Due to privacy concerns surrounding patient data, we cannot transmit profiles outside the institution’s firewall. To address this limitation, we examined four locally hosted LLMs. The first two, GPT-3.5 Turbo and GPT-4^11^, were accessed through a Microsoft Azure service. The third, Llama-2 from Meta^12^, was obtained via Hugging Face and run on an Amazon EC2 machine with 4 NVIDIA A10G Tensor Core GPUs. However, the initial version of Llama-2 exhibited poor performance. Consequently, we opted for Sheep-Duck-Llama-2, a modified version of Llama-2 fine-tuned on Orca-style and Alpaca-style datasets, that scored highest on the Huggingface LLM Leaderbord at the time^13–15^. For all LLMs we used a temperature of 0.

### Prompt engineering

We mainly focus on zero-shot prompting^16^, meaning we do not include any examples when prompting the LLMs. The output from KEEPER was turned into text by grouping categories together. For instance, disease history, symptoms, comorbidities, and risk factors were combined into a single category ‘Diagnoses recorded prior to the visit’. See the Supplementary Table 2 for the groupings of categories.

The prompt featuring the KEEPER output was accompanied by a system prompt guiding the LLM on how to process the information. We employed an ad-hoc approach to prompt engineering. Initially, we used a straightforward system prompt instructing the LLM to provide a binary ‘yes’ or ‘no’ answer regarding whether the patient had the specified disease. We iteratively expanded the system prompt based on the LLM’s performance and errors observed during runs on the development set. All iterations and performance metrics are reported in the Results section, and were performed with GPT-4 alone, as it proved to be the most efficient system. The code for generating the various prompts is available in the KEEPER R package. (https://github.com/OHDSI/Keeper/tree/v0.2.0). The responses of the LLMs are parsed using a simple approach, removing all but the ‘Summary’ section and looking for keywords such as ‘yes’ and ‘no’. (See Supplementary Note 3) For computation of the evaluation metrics, a failure to choose between ‘yes’ and ‘no’ was interpreted as ‘no’.

Our evaluation metrics included sensitivity, specificity, and AUC with the gold standard (human annotation). Because both human and LLM reviewer produce binary outputs, we can compute AUC as the mean of the sensitivity and the specificity.

### Data sources

We used two data sources: Columbia University Irving Medical (CUIMC) EHRs and the Optum’s de-identified Clinformatics® Data Mart Database (Clinformatics®).

CUIMC EHRs translated to the OMOP CDM, comprising electronic health records and data from administrative and ancillary systems for over six million patients. The database encompasses person details, visit information (inpatient and outpatient), conditions (billing diagnoses and problem lists), drugs (outpatient prescriptions and inpatient orders/administrations), devices, measurements (laboratory tests and vital signs), and other observations (symptoms). IRB for original KEEPER: Columbia University Medical center institutional review board (IRB-AAAS6414).

Clinformatics®, also translated to the OMOP CDM, contains administrative health claims for members in large commercial and Medicare Advantage health plans. It encompasses over 65 million unique patients, providing patient-level data from claims related to enrollment, person details, drug dispensing, procedures, diagnoses, and admission and discharge dates. Approximately 30% of the laboratory tests are recorded with the results. The population is geographically diverse, representing 50 of the United States of America. The use of Clinformatics® was reviewed by the New England Institutional Review Board (IRB) and was determined to be exempt from broad IRB approval, as this research project did not involve human subject research.

### Construction of development and test sets

**Fig. 4:**
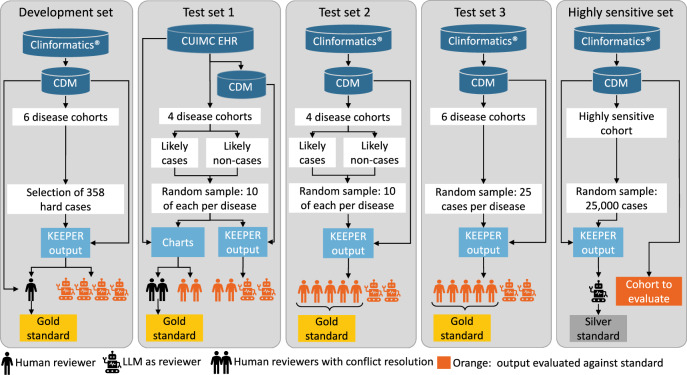
Overview of development and test sets used in this study. A development set was created to guide prompt engineering. Test set 1 was also used in our prior work on KEEPER, thus providing a benchmark for consistency. Test set 2 mimics 1 but uses insurance claims data. Test set 3 takes a truly random sample across more diseases to enhance generalizability. The highly sensitive set demonstrates the use of LLMs to annotate a large set of patients, allowing computation of sensitivity and PPV of phenotype algorithms.

A total of 5 sets with corresponding gold or silver standard case labels were created, as detailed in Fig. 4. See Supplementary Table 3 for case definitions, Supplementary Note 1 for phenotype algorithms, and Supplementary Note 2 for KEEPER concept sets. All human reviewers were board-certified clinicians.

To develop our prompting strategy, we first created a development set encompassing six diseases: acute bronchitis, hyperlipidemia, hypoparathyroidism, osteoporosis, RA, and viral hepatitis type A. These six diseases were chosen to represent a diverse spectrum of therapeutic areas. Case definitions and phenotype algorithms, detailed in Supplementary Table 3 and Supplementary Note 1, respectively, were employed to identify cases within the Clinformatics® database. A non-random sample of 358 patients was selected by manual review, emphasizing challenging cases (cases that neither had all markers of a case nor no markers). The gold standard was created by a single reviewer using the KEEPER output as well as any other data available in the CDM.

Following the selection of our final prompting strategy, we applied it to three distinct test sets:

1. Test Set 1, taken from the original KEEPER paper^3^:

- Comprising four diseases: acute appendicitis, diabetes mellitus type I, chronic obstructive pulmonary disorder (COPD), and end-stage renal disease.
- Balanced sample (half—likely cases and half—likely non-cases) of 20 cases per disease from the CUIMC database. Likely cases were defined as those having more restrictive inclusion criteria such as a diagnosis of appendicitis followed by antibiotic therapy or appendectomy and likely-non cases were defined through less restrictive phenotypes such as an occurrence of an appendicitis code.
- Annotated by two reviewers, each utilizing both KEEPER and full charts separately, resulting in 4 times 80 case adjudications.
- Gold standard was two independent reviewers performing chart review using all available structured and unstructured data. Results were discussed and iterative chart review continued until all disagreements were resolved.

2. Test Set 2:

- Same diseases as Test Set 1.
- Balanced sample (half—likely cases and half—likely non-cases) of 20 cases per disease in the Clinformatics® database.
- Independently adjudicated by five reviewers using KEEPER.
- Gold standard was the majority vote of human reviewers, leaving a reviewer out of the vote when evaluating that reviewer.

3. Test Set 3:

- Comprising the six diseases from the development set.
- Random sample of 25 cases per disease from the Clinformatics® database.
- Independently adjudicated by five reviewers using KEEPER.
- Gold standard was the majority vote of human reviewers, leaving a reviewer out of the vote when evaluating that reviewer.

Additionally, we create a final set without human review to demonstrate the feasibility of adjudicating a high-sensitivity cohort. A high-sensitivity cohort is designed to have near-perfect sensitivity, likely at the cost of very low specificity, and has been suggested as a way to make adjudication of both cases and non-cases feasible when the outcome is rare^2^. Specifically, we construct a high-sensitive cohort for RA, including all patients with any relevant diagnose, symptom, treatment, complication, or laboratory test, and take a random sample of 25,000 patients. GPT-4, using KEEPER annotates the 25,000 as cases and non-cases, allowing the set to be used to compute PPV and sensitivity for any phenotype algorithm. We demonstrate its use on an established phenotype algorithm for RA from the OHDSI Phenotype Library.

## Supplementary information

Supplemental Materials

## Data Availability

The patient-level data used in this study cannot be shared, for privacy and licensing reasons. Others could also license the Clinformatics® data from Optum®.

## Code availability

The code used in this study is made available in Supplementary Note 3 and online in the KEEPER R package: https://github.com/OHDSI/Keeper/tree/v0.2.0.
