# Supplementary material for: Standardized patient profile review using large language models for case adjudication in observational research
Source: NPJ Digit Med. 2025 Jan 9;8:18. doi: 10.1038/s41746-025-01433-4 (PMC11718233; doi:10.1038/s41746-025-01433-4)
Supplement: Supplementary file 1 — Supplemental Materials [file 41746_2025_1433_MOESM1_ESM.pdf]

Supplementary Materials

For "Standardized patient profile review using large language models for case adjudication in observational research"

Table of Contents

Supplementary Table 1: KEEPER time windows 2
Supplementary Table 2: Groupings of KEEPER categories 3
Supplementary Table 3 : Outcome case definitions 3
Supplementary Note 1: Outcome Phenotype Algorithms 4
Rheumatoid Arthritis (Cohort 858) 4
Cohort Entry Events 4
Cohort Exit 4
Cohort Eras 4
Rheumatoid arthritis 4
Hyperlipidemia (Cohort 1219) 5
Cohort Entry Events 5
Cohort Exit 5
Cohort Eras 5
Hyperlipidemia 5
Hypoparathyroidism (Cohort 1220) 5
Cohort Entry Events 5
Cohort Exit 5
Cohort Eras 6
Hypoparathyroidism 6
Osteoporosis (Cohort 1221) 6
Cohort Entry Events 6
Cohort Exit 6
Cohort Eras 6
Osteoporosis 6
Viral Hepatitis A (Cohort 1222) 6
Cohort Entry Events 6
Cohort Exit 6
Cohort Eras 6

Type A viral hepatitis 7
Supplementary Note 2: KEEPER input concept sets 7
Acute bronchitis 7
Hyperlipidemia 8
Hypoparathyroidism 11
Osteoporosis 17
Rheumatoid arthritis 20
Viral hepatitis type A 24
Diabetes mellitus type I 31
Acute appendicitis 34
End-stage renal disease 45
Chronic obstructive pulmonary disorder (COPD) 47
Supplementary Note 3: LLM response parser 48
Supplementary Note 4: System prompt templates 49
Yes/no 49
+ discuss evidence 50
+ write narrative 50
+ diagnosis insufficient reminder 50
+ uncertainty instructions 51
+ provide examples 51
Supplementary Note 5: Example prompt 54
Supplementary Note 6: Example LLM output 54
Supplementary Note 7: Performance on test set 1 56
Supplementary Note 8: Performance on test set 2 58
Supplementary Note 9: Performance on test set 3 60

Supplementary Table 1: KEEPER time windows

Category	Days relative to index date
Demographics	0
Presentation	0
Disease history	-9999 to -1
Disease progression	1 to 9999
Symptoms	-30 to -1
Comorbidities and risk factors	-9999 to -1

Category	Days relative to index date
Differential diagnoses	-90 to 90
Diagnostic procedures	-30 to 30
Measurements	-9999 to -1
Drug treatment history	-9999 to -1
Drug treatment	0 to 9999
Procedure treatment history	-9999 to -1
Procedure treatment	0 to 9999
Death	0 to 9999
Visit context	0

Supplementary Table 2: Groupings of KEEPER categories

To generate the prompt, KEEPER categories were combined into single sections and provided with a descriptive header.

Section header	KEEPER sections
Demographics and details about the visit:	demographics, visit context
Diagnoses recorded on the day of the visit:	presentation
Diagnoses recorded prior to the visit:	disease history, symptoms, comorbidities, risk factors
Treatments recorded prior to the visit:	drug treatment history, procedure treatment history
Diagnostic procedures recorded proximal to the visit:	diagnostic procedures
Laboratory tests recorded proximal to the visit:	measurements
Alternative diagnoses recorded proximal to the visit:	differential diagnoses
Diagnoses recorded after the visit:	disease progression
Treatments recorded during or after the visit:	drug treatment, procedure treatment

Supplementary Table 3 : Outcome case definitions

The outcome case definitions used in this study are those provided by the National Library of Medicine. The table below lists the hyperlinks to the relevant pages for each disease.

Disease	Clinical description link
Acute bronchitis.	https://www.ncbi.nlm.nih.gov/books/NBK448067/
Hyperlipidemia.	https://www.ncbi.nlm.nih.gov/books/NBK559182/
Hypoparathyroidism.	https://www.ncbi.nlm.nih.gov/books/NBK441899/
Osteoporosis.	https://www.ncbi.nlm.nih.gov/books/NBK441901/
Rheumatoid arthritis.	https://www.ncbi.nlm.nih.gov/books/NBK441999/
Viral hepatitis type A	https://www.ncbi.nlm.nih.gov/books/NBK459290/

Supplementary Note 1: Outcome Phenotype Algorithms

The phenotype algorithms used in this study are part of the OHDSI Phenotype Library (https://ohdsi.github.io/PhenotypeLibrary/), where computer-executable SQL code can be found to execute the algorithms on a database in the OMOP Common Data Model. The Cohort ID mentioned in each section header corresponds to the ID in the Phenotype Library.

Rheumatoid Arthritis (Cohort 858)

Cohort Entry Events

People enter the cohort when observing any of the following:

1. condition occurrence of 'Rheumatoid arthritis' for the first time in the person's history.
2. observation of 'Rheumatoid arthritis' for the first time in the person's history.

Limit cohort entry events to the earliest event per person.

Cohort Exit

The person also exists the cohort at the end of continuous observation.

Cohort Eras

Remaining events will be combined into cohort eras if they are within 0 days of each other.

Rheumatoid arthritis

Concept ID	Concept Name	Code	Vocabulary	Excluded	Descendants	Mapped
4297650	Cutaneous atrophy due to rheumatoid arthritis	402431009	SNOMED	NO	YES	NO
4334806	Deformity of foot due to rheumatoid arthritis	433228003	SNOMED	NO	YES	NO
46273442	Deformity of hand due to rheumatoid arthritis	96531000119109	SNOMED	NO	YES	NO
2107572	Disease prognosis for rheumatoid arthritis assessed, good prognosis documented (RA)	3476F	CPT4	NO	YES	NO
2107561	Disease prognosis for rheumatoid arthritis assessed, poor prognosis documented (RA)	3475F	CPT4	NO	YES	NO
4107913	Myopathy due to rheumatoid arthritis	193250002	SNOMED	NO	YES	NO
2108721	Patient receiving first-time biologic disease modifying anti-rheumatic drug therapy for rheumatoid arthritis (RA)	4195F	CPT4	NO	YES	NO
4102493	Polyneuropathy in rheumatoid arthritis	193180002	SNOMED	NO	YES	NO
80809	Rheumatoid arthritis	69896004	SNOMED	NO	YES	NO
2107560	Rheumatoid arthritis (RA) disease	3472F	CPT4	NO	YES	NO

Concept ID	Concept Name	Code	Vocabulary	Excluded	Descendants	Mapped
	activity, high (RA)					
2107558	Rheumatoid arthritis (RA) disease activity, low (RA)	3470F	CPT4	NO	YES	NO
2107559	Rheumatoid arthritis (RA) disease activity, moderate (RA)	3471F	CPT4	NO	YES	NO
4083556	Seronegative rheumatoid arthritis	239792003	SNOMED	NO	YES	NO
4035611	Seropositive rheumatoid arthritis	239791005	SNOMED	NO	YES	NO

Hyperlipidemia (Cohort 1219)

Cohort Entry Events

People enter the cohort when observing any of the following:

1. condition occurrences of 'Hyperlipidemia'.

Limit cohort entry events to the earliest event per person.

Cohort Exit

The person also exists the cohort at the end of continuous observation.

Cohort Eras

Remaining events will be combined into cohort eras if they are within 0 days of each other.

Hyperlipidemia

Concept ID	Concept Name	Code	Vocabulary	Excluded	Descendants	Mapped
432867	Hyperlipidemia	55822004	SNOMED	NO	YES	NO
437530	Disorder of lipid metabolism	267431006	SNOMED	NO	NO	NO

Hypoparathyroidism (Cohort 1220)

Cohort Entry Events

People enter the cohort when observing any of the following:

1. condition occurrences of 'Hypoparathyroidism'.

Limit cohort entry events to the earliest event per person.

Cohort Exit

The person also exists the cohort at the end of continuous observation.

Cohort Eras

Remaining events will be combined into cohort eras if they are within 0 days of each other.

Hypoparathyroidism

Concept ID	Concept Name	Code	Vocabulary	Excluded	Descendants	Mapped
140362	Hypoparathyroidism	36976004	SNOMED	NO	YES	NO

Osteoporosis (Cohort 1221)

Cohort Entry Events

People enter the cohort when observing any of the following:

1. condition occurrences of 'Osteoporosis'.

Limit cohort entry events to the earliest event per person.

Cohort Exit

The person also exists the cohort at the end of continuous observation.

Cohort Eras

Remaining events will be combined into cohort eras if they are within 0 days of each other.

Osteoporosis

Concept ID	Concept Name	Code	Vocabulary	Excluded	Descendants	Mapped
80502	Osteoporosis	64859006	SNOMED	NO	YES	NO

Viral Hepatitis A (Cohort 1222)

Cohort Entry Events

People enter the cohort when observing any of the following:

1. condition occurrences of 'Type A viral hepatitis'.

Limit cohort entry events to the earliest event per person.

Cohort Exit

The person also exists the cohort at the end of continuous observation.

Cohort Eras

Remaining events will be combined into cohort eras if they are within 0 days of each other.

Type A viral hepatitis

Concept ID	Concept Name	Code	Vocabulary	Excluded	Descendants	Mapped
4223947	Viral hepatitis, type A	40468003	SNOMED	NO	YES	NO
4013974	Hepatitis A test positive	165997004	SNOMED	NO	YES	NO
194856	Hepatic coma due to viral hepatitis A	16060001	SNOMED	NO	YES	NO

Supplementary Note 2: KEEPER input concept sets

The KEEPER algorithm automatically expands the concept sets below to include all descendant concepts.

Acute bronchitis

KEEPER category	Concept ID	Concept Name
Disease of Interest	255841	Chronic bronchitis
Disease of Interest	256451	Bronchitis
Disease of Interest	260139	Acute bronchitis
Measurements	4020553	Oxygen saturation measurement
Measurements	4220748	Immunoglobulin E measurement
Complications	255848	Pneumonia
Complications	4195694	Acute respiratory distress syndrome
Complications	4256228	Respiratory failure
Alternative Diagnosis	255573	Chronic obstructive lung disease
Alternative Diagnosis	257011	Acute upper respiratory infection
Alternative Diagnosis	260123	Acute sinusitis
Alternative Diagnosis	316139	Heart failure
Alternative Diagnosis	317009	Asthma
Alternative Diagnosis	318800	Gastroesophageal reflux disease
Alternative Diagnosis	440417	Pulmonary embolism
Alternative	4035987	Viral pharyngitis

KEEPER category	Concept ID	Concept Name
Diagnosis		
Alternative Diagnosis	4165112	Bronchiolitis
Symptoms	254761	Cough
Symptoms	256451	Bronchitis
Symptoms	260123	Acute sinusitis
Symptoms	4047791	Abnormal body temperature
Symptoms	4089228	Sputum finding
Symptoms	46273539	Acute bronchospasm
Comorbidities	437264	Tobacco dependence syndrome
Comorbidities	443410	Infective pneumonia
Drugs	1101703	homatropine
Drugs	1136601	benzonatate
Drugs	1153428	fexofenadine
Drugs	1154343	albuterol
Drugs	1163944	guaifenesin
Drugs	1192218	levalbuterol
Drugs	1192710	chlorpheniramine
Drugs	1713332	amoxicillin
Drugs	1734104	azithromycin
Drugs	1750500	clarithromycin
Diagnostic Procedures	2008419	Other miscellaneous procedures
Diagnostic Procedures	2211361	Radiologic examination, chest, 2 views, frontal and lateral
Diagnostic Procedures	2314026	Pressurized or nonpressurized inhalation treatment for acute airway obstruction for therapeutic purposes and/or for diagnostic purposes such as sputum induction with an aerosol generator, nebulizer, metered dose inhaler or intermittent positive pressure b
Diagnostic Procedures	2314037	Demonstration and/or evaluation of patient utilization of an aerosol generator, nebulizer, metered dose inhaler or IPPB device
Diagnostic Procedures	42738270	Blood count; hemogram and platelet count, automated, and automated partial differential WBC count (CBC) (Deprecated)
Diagnostic Procedures	42740865	Therapeutic, prophylactic or diagnostic injection (specify substance or drug); subcutaneous or intramuscular

Hyperlipidemia

KEEPER category	Concept ID	Concept Name
Disease of Interest	432867	Hyperlipidemia
Disease of Interest	437530	Disorder of lipid metabolism
Alternative	437530	Disorder of lipid metabolism

KEEPER category	Concept ID	Concept Name
Diagnosis		
Comorbidities	77646	Disorder of bone and articular cartilage
Comorbidities	81611	Diverticular disease of colon
Comorbidities	201072	Benign prostatic hyperplasia without outflow obstruction
Comorbidities	201820	Diabetes mellitus
Comorbidities	312648	Benign essential hypertension
Comorbidities	317576	Coronary arteriosclerosis
Comorbidities	318800	Gastroesophageal reflux disease
Comorbidities	319843	Mitral valve disorder
Comorbidities	432867	Hyperlipidemia
Comorbidities	437264	Tobacco dependence syndrome
Comorbidities	437530	Disorder of lipid metabolism
Comorbidities	438557	Abnormal glucose level
Comorbidities	443238	Diabetic - poor control
Comorbidities	4172857	Menopause finding
Complications	134057	Disorder of cardiovascular system
Drugs	1510813	rosuvastatin
Drugs	1539403	simvastatin
Drugs	1545958	atorvastatin
Drugs	1549686	fluvastatin
Drugs	1551860	pravastatin
Drugs	1592085	lovastatin
Drugs	1592180	cerivastatin
Drugs	1797258	cilastatin
Drugs	40165636	pitavastatin
Measurements	3000905	Leukocytes [#/volume] in Blood by Automated count
Measurements	3000963	Hemoglobin [Mass/volume] in Blood
Measurements	3004501	Glucose [Mass/volume] in Serum or Plasma
Measurements	3006906	Calcium [Mass/volume] in Serum or Plasma
Measurements	3006923	Alanine aminotransferase [Enzymatic activity/volume] in Serum or Plasma
Measurements	3007070	Cholesterol in HDL [Mass/volume] in Serum or Plasma
Measurements	3009220	Epithelial cells.squamous [#/volume] in Urine sediment
Measurements	3009596	Cholesterol in VLDL [Mass/volume] in Serum or Plasma by calculation
Measurements	3009744	MCHC [Mass/volume] by Automated count
Measurements	3012030	MCH [Entitic mass] by Automated count
Measurements	3013473	Cholesterol in HDL [Mass/volume] in Serum or Plasma ultracentrifugate
Measurements	3013682	Urea nitrogen [Mass/volume] in Serum or Plasma
Measurements	3013721	Aspartate aminotransferase [Enzymatic activity/volume] in Serum or Plasma
Measurements	3014576	Chloride [Moles/volume] in Serum or Plasma

KEEPER category	Concept ID	Concept Name
Measurements	3015632	Carbon dioxide, total [Moles/volume] in Serum or Plasma
Measurements	3016723	Creatinine [Mass/volume] in Serum or Plasma
Measurements	3018311	Urea nitrogen/Creatinine [Mass Ratio] in Serum or Plasma
Measurements	3019550	Sodium [Moles/volume] in Serum or Plasma
Measurements	3019897	Erythrocyte distribution width [Ratio] by Automated count
Measurements	3020416	Erythrocytes [#/volume] in Blood by Automated count
Measurements	3020509	Albumin/Globulin [Mass Ratio] in Serum or Plasma
Measurements	3020630	Protein [Mass/volume] in Serum or Plasma
Measurements	3022113	Urinalysis microscopic panel - Urine sediment
Measurements	3022192	Triglyceride [Mass/volume] in Serum or Plasma
Measurements	3023103	Potassium [Moles/volume] in Serum or Plasma
Measurements	3023314	Hematocrit [Volume Fraction] of Blood by Automated count
Measurements	3023599	MCV [Entitic volume] by Automated count
Measurements	3024128	Bilirubin.total [Mass/volume] in Serum or Plasma
Measurements	3024561	Albumin [Mass/volume] in Serum or Plasma
Measurements	3024929	Platelets [#/volume] in Blood by Automated count
Measurements	3027114	Cholesterol [Mass/volume] in Serum or Plasma
Measurements	3027970	Globulin [Mass/volume] in Serum by calculation
Measurements	3028089	Alkaline phosphatase isoenzyme [Units/volume] in Serum or Plasma
Measurements	3028288	Cholesterol in LDL [Mass/volume] in Serum or Plasma by calculation
Measurements	3035995	Alkaline phosphatase [Enzymatic activity/volume] in Serum or Plasma
Measurements	3040168	Immature granulocytes [#/volume] in Blood
Measurements	4018619	Screening for occult blood in feces
Measurements	4034850	Laboratory test
Measurements	4062484	Screening for malignant neoplasm of cervix
Measurements	4077697	Screening mammography
Measurements	4152047	Hyperlipidemia screening
Measurements	4175226	Screening for malignant neoplasm of colon
Measurements	4246879	Thyroid stimulating hormone measurement
Measurements	40481096	Screening for malignant neoplasm of prostate
Measurements	44793131	Total prostate specific antigen level
Diagnostic Procedures	9201	Inpatient Visit
Diagnostic Procedures	2001500	Single vessel percutaneous transluminal coronary angioplasty [PTCA] or coronary atherectomy without mention of thrombolytic agent
Diagnostic Procedures	2008419	Other miscellaneous procedures
Diagnostic Procedures	2108115	Collection of venous blood by venipuncture
Diagnostic Procedures	2211359	Radiologic examination, chest; single view, frontal

KEEPER category	Concept ID	Concept Name
Diagnostic Procedures	2211361	Radiologic examination, chest, 2 views, frontal and lateral
Diagnostic Procedures	2313814	Electrocardiogram, routine ECG with at least 12 leads; with interpretation and report
Diagnostic Procedures	2313870	Echocardiography, transthoracic, real-time with image documentation (2D), includes M-mode recording, when performed, complete, without spectral or color Doppler echocardiography
Diagnostic Procedures	4036803	General examination of patient
Diagnostic Procedures	4141759	Long-term drug therapy
Diagnostic Procedures	4283892	Placement of stent in coronary artery
Diagnostic Procedures	4306780	Gynecologic examination
Diagnostic Procedures	42734963	Myocardial perfusion study with wall motion, qualitative or quantitative study (List separately in addition to code for primary procedure)
Diagnostic Procedures	42734964	Myocardial perfusion study with ejection fraction (List separately in addition to code for primary procedure)
Diagnostic Procedures	42737560	Screening mammography, bilateral (two view film study of each breast) (Deprecated)
Diagnostic Procedures	42738270	Blood count; hemogram and platelet count, automated, and automated partial differential WBC count (CBC) (Deprecated)
Diagnostic Procedures	42738308	Unlisted miscellaneous pathology test (Deprecated)
Diagnostic Procedures	42739106	Myocardial perfusion imaging; tomographic (SPECT), multiple studies (including attenuation correction when performed), at rest and/or stress (exercise and/or pharmacologic) and redistribution and/or rest injection, with or without quantification
Diagnostic Procedures	42739299	Lipoprotein, blood; high resolution fractionation and quantitation of lipoprotein cholesterols (eg, electrophoresis, nuclear magnetic resonance, ultracentrifugation) (Deprecated)
Diagnostic Procedures	44782057	DRAWING BLOOD FOR SPECIMEN (Deprecated)

Hypoparathyroidism

KEEPER category	Concept ID	Concept Name
Disease of Interest	140362	Hypoparathyroidism
Alternative Diagnosis	77646	Disorder of bone and articular cartilage
Alternative Diagnosis	140357	Neoplasm of uncertain behavior of endocrine gland
Alternative Diagnosis	255919	Finding of head and neck region

KEEPER category	Concept ID	Concept Name
Alternative Diagnosis	315085	Lymphadenopathy
Alternative Diagnosis	380534	Tetany
Alternative Diagnosis	434300	Neuroendocrine tumor
Alternative Diagnosis	435788	Disorder of phosphorus metabolism
Alternative Diagnosis	437828	Disorder of calcium metabolism
Alternative Diagnosis	438624	Complication of renal dialysis
Alternative Diagnosis	441830	Disorder of fluid AND/OR electrolyte
Alternative Diagnosis	444239	Postprocedural state finding
Alternative Diagnosis	4130839	Neoplasm of lymph node
Alternative Diagnosis	4172857	Menopause finding
Alternative Diagnosis	4176807	Endocrine system complication of procedure
Alternative Diagnosis	35624485	22q11.2 deletion syndrome
Alternative Diagnosis	42537310	Neonatal metabolic disorder
Alternative Diagnosis	45770748	Endocrine function test abnormal
Complications	435510	Hypocalcemia
Symptoms	30365	Panhypopituitarism
Symptoms	77646	Disorder of bone and articular cartilage
Symptoms	133727	Thyrotoxicosis without goiter or other cause
Symptoms	135215	Hashimoto thyroiditis
Symptoms	138384	Acquired hypothyroidism
Symptoms	138713	Disorder of parathyroid gland
Symptoms	140364	Disorder of thyrocalcitonin secretion
Symptoms	435788	Disorder of phosphorus metabolism
Symptoms	437828	Disorder of calcium metabolism
Symptoms	438624	Complication of renal dialysis
Symptoms	439926	Malaise and fatigue
Symptoms	441830	Disorder of fluid AND/OR electrolyte
Symptoms	444239	Postprocedural state finding
Symptoms	4090425	Altered sensation of skin

KEEPER category	Concept ID	Concept Name
Symptoms	4150384	Urine cytology abnormal
Comorbidities	30365	Panhypopituitarism
Comorbidities	77646	Disorder of bone and articular cartilage
Comorbidities	133424	Primary malignant neoplasm of thyroid gland
Comorbidities	133727	Thyrotoxicosis without goiter or other cause
Comorbidities	133728	Congenital hypothyroidism
Comorbidities	135215	Hashimoto thyroiditis
Comorbidities	135772	Goiter
Comorbidities	138384	Acquired hypothyroidism
Comorbidities	140357	Neoplasm of uncertain behavior of endocrine gland
Comorbidities	319843	Mitral valve disorder
Comorbidities	380534	Tetany
Comorbidities	438624	Complication of renal dialysis
Comorbidities	444239	Postprocedural state finding
Comorbidities	4113821	Anxiety state
Comorbidities	4130839	Neoplasm of lymph node
Comorbidities	4172857	Menopause finding
Comorbidities	35624485	22q11.2 deletion syndrome
Comorbidities	44782690	Chronic kidney disease stage 5 due to hypertension
Measurements	2212090	Basic metabolic panel (Calcium, total) This panel must include the following: Calcium, total (82310) Carbon dioxide (bicarbonate) (82374) Chloride (82435) Creatinine (82565) Glucose (82947) Potassium (84132) Sodium (84295) Urea nitrogen (BUN) (84520)
Measurements	2212186	Albumin; serum, plasma or whole blood
Measurements	2212241	Calcium; total
Measurements	2212244	Calcium; urine quantitative, timed specimen
Measurements	2212284	Cortisol; total
Measurements	2212601	Thyroid hormone (T3 or T4) uptake or thyroid hormone binding ratio (THBR)
Measurements	2213346	Unlisted miscellaneous pathology test
Measurements	3000067	Parathyrin.intact [Mass/volume] in Serum or Plasma
Measurements	3006906	Calcium [Mass/volume] in Serum or Plasma
Measurements	3014173	Calcitriol [Mass/volume] in Serum or Plasma
Measurements	3015377	Calcium [Moles/volume] in Serum or Plasma
Measurements	3018311	Urea nitrogen/Creatinine [Mass Ratio] in Serum or Plasma
Measurements	3020029	Reducing substances [Presence] in Urine
Measurements	3024250	Calcium.ionized [Mass/volume] in Serum or Plasma by Ion-selective membrane electrode (ISE)
Measurements	3025547	Thyroglobulin Ab [Units/volume] in Serum or Plasma
Measurements	4007831	Vitamin D, 25-hydroxy measurement
Measurements	4017908	Phosphate, inorganic measurement

KEEPER category	Concept ID	Concept Name
Measurements	4034850	Laboratory test
Measurements	4141751	Parathyroid hormone measurement
Measurements	4147378	Calcitonin measurement
Measurements	4196731	Total thyroxine measurement
Measurements	4196969	T4 free measurement
Measurements	4219068	Vitamin D, 1,25-dihydroxy measurement
Measurements	4219426	Thyroglobulin measurement
Measurements	4243005	Magnesium measurement
Measurements	4246879	Thyroid stimulating hormone measurement
Measurements	46272910	Measurement of ionized calcium in blood specimen
Diagnostic Procedures	9201	Inpatient Visit
Diagnostic Procedures	581477	Office Visit
Diagnostic Procedures	2000265	Closed [percutaneous] [needle] biopsy of thyroid gland
Diagnostic Procedures	2000269	Unilateral thyroid lobectomy
Diagnostic Procedures	2000272	Other partial thyroidectomy
Diagnostic Procedures	2000282	Other parathyroidectomy
Diagnostic Procedures	2001292	Laryngoscopy and other tracheoscopy
Diagnostic Procedures	2002307	Simple excision of other lymphatic structure
Diagnostic Procedures	2002311	Radical neck dissection, unilateral
Diagnostic Procedures	2007488	Other radiotherapeutic procedure
Diagnostic Procedures	2008419	Other miscellaneous procedures
Diagnostic Procedures	2100740	Anesthesia for all procedures on esophagus, thyroid, larynx, trachea and lymphatic system of neck; not otherwise specified, age 1 year or older
Diagnostic Procedures	2101871	Fine needle aspiration; with imaging guidance
Diagnostic Procedures	2106527	Laryngoscopy, flexible; diagnostic
Diagnostic Procedures	2108115	Collection of venous blood by venipuncture
Diagnostic Procedures	2108148	Venous catheterization for selective organ blood sampling
Diagnostic Procedures	2108471	Dissection, deep jugular node(s)

KEEPER category	Concept ID	Concept Name
Diagnostic Procedures	2108482	Cervical lymphadenectomy (modified radical neck dissection)
Diagnostic Procedures	2110365	Excision of cyst or adenoma of thyroid, or transection of isthmus
Diagnostic Procedures	2110367	Partial thyroid lobectomy, unilateral; with or without isthmusectomy
Diagnostic Procedures	2110369	Total thyroid lobectomy, unilateral; with or without isthmusectomy
Diagnostic Procedures	2110370	Total thyroid lobectomy, unilateral; with contralateral subtotal lobectomy, including isthmusectomy
Diagnostic Procedures	2110372	Thyroidectomy, total or subtotal for malignancy; with limited neck dissection
Diagnostic Procedures	2110373	Thyroidectomy, total or subtotal for malignancy; with radical neck dissection
Diagnostic Procedures	2110374	Thyroidectomy, removal of all remaining thyroid tissue following previous removal of a portion of thyroid
Diagnostic Procedures	2110376	Thyroidectomy, including substernal thyroid; cervical approach
Diagnostic Procedures	2110383	Parathyroidectomy or exploration of parathyroid(s)
Diagnostic Procedures	2110384	Parathyroidectomy or exploration of parathyroid(s); re-exploration
Diagnostic Procedures	2110386	Parathyroid autotransplantation (List separately in addition to code for primary procedure)
Diagnostic Procedures	2110387	Thymectomy, partial or total; transcervical approach (separate procedure)
Diagnostic Procedures	2110396	Unlisted procedure, endocrine system
Diagnostic Procedures	2110820	Neuroplasty and/or transposition; cranial nerve (specify)
Diagnostic Procedures	2211337	Computed tomography, soft tissue neck; with contrast material(s)
Diagnostic Procedures	2211359	Radiologic examination, chest; single view, frontal
Diagnostic Procedures	2211361	Radiologic examination, chest, 2 views, frontal and lateral
Diagnostic Procedures	2211727	Unlisted diagnostic radiographic procedure
Diagnostic Procedures	2211737	Ultrasound, soft tissues of head and neck (eg, thyroid, parathyroid, parotid), real time with image documentation
Diagnostic Procedures	2211906	Supervision, handling, loading of radiation source
Diagnostic Procedures	2211909	Thyroid uptake; single determination
Diagnostic	2211912	Thyroid imaging, with uptake; single determination

KEEPER category	Concept ID	Concept Name
Procedures		
Diagnostic Procedures	2211913	Thyroid imaging, with uptake; multiple determinations
Diagnostic Procedures	2211914	Thyroid imaging; only
Diagnostic Procedures	2211918	Thyroid carcinoma metastases imaging; whole body
Diagnostic Procedures	2211919	Thyroid carcinoma metastases uptake (List separately in addition to code for primary procedure)
Diagnostic Procedures	2211920	Parathyroid planar imaging (including subtraction, when performed)
Diagnostic Procedures	2212051	Injection procedure for radiopharmaceutical localization by non-imaging probe study, intravenous (eg, parathyroid adenoma)
Diagnostic Procedures	2212066	Radiopharmaceutical therapy, by oral administration
Diagnostic Procedures	2212080	Radiopharmaceutical therapy, unlisted procedure
Diagnostic Procedures	2213284	Level V - Surgical pathology, gross and microscopic examination Adrenal, resection Bone - biopsy/curettings Bone fragment(s), pathologic fracture Brain, biopsy Brain/meninges, tumor resection Breast, excision of lesion, requiring microscopic evaluation of
Diagnostic Procedures	2213296	Pathology consultation during surgery; first tissue block, with frozen section(s), single specimen
Diagnostic Procedures	2313870	Echocardiography, transthoracic, real-time with image documentation (2D), includes M-mode recording, when performed, complete, without spectral or color Doppler echocardiography
Diagnostic Procedures	2314122	Needle electromyography; larynx
Diagnostic Procedures	2314125	Needle electromyography; cranial nerve supplied muscles, bilateral
Diagnostic Procedures	2314135	Intraoperative neurophysiology testing, per hour (List separately in addition to code for primary procedure)
Diagnostic Procedures	2740457	Resection of Right Neck Lymphatic, Open Approach
Diagnostic Procedures	2740654	Resection of Left Neck Lymphatic, Open Approach
Diagnostic Procedures	2749795	Resection of Left Thyroid Gland Lobe, Open Approach
Diagnostic Procedures	2749797	Resection of Right Thyroid Gland Lobe, Open Approach
Diagnostic Procedures	4004516	Pre-surgery evaluation
Diagnostic Procedures	4064257	Excision of regional lymph nodes
Diagnostic Procedures	4073199	Total thyroidectomy

KEEPER category	Concept ID	Concept Name
Diagnostic Procedures	4120120	Hemodialysis
Diagnostic Procedures	4141759	Long-term drug therapy
Diagnostic Procedures	4197217	Arteriovenous anastomosis for renal dialysis
Diagnostic Procedures	4231441	Excision of deep cervical lymph nodes
Diagnostic Procedures	4233827	Biopsy of lymphatic structure
Diagnostic Procedures	4289454	Venous catheterization for renal dialysis
Diagnostic Procedures	4306780	Gynecologic examination
Diagnostic Procedures	40480674	Preoperative cardiovascular examination
Diagnostic Procedures	42734948	Radiopharmaceutical ablation of gland for thyroid carcinoma (Deprecated)
Diagnostic Procedures	42736574	Introduction of needle or intracatheter; arteriovenous shunt created for dialysis (cannula, fistula, or graft)
Diagnostic Procedures	42737560	Screening mammography, bilateral (two view film study of each breast) (Deprecated)
Diagnostic Procedures	42737977	Angiography, arteriovenous shunt (eg, dialysis patient), radiological supervision and interpretation
Diagnostic Procedures	42738268	Blood count; hemogram, automated, and manual differential WBC count (CBC) (Deprecated)
Diagnostic Procedures	42738308	Unlisted miscellaneous pathology test (Deprecated)
Diagnostic Procedures	44782057	DRAWING BLOOD FOR SPECIMEN (Deprecated)
Drugs	836208	succinylcholine
Drugs	1125315	acetaminophen
Drugs	1501700	levothyroxine
Drugs	1505346	liothyronine
Drugs	19007721	thyrotropin
Drugs	19035631	calcitriol
Drugs	19037038	calcium gluconate
Drugs	19049909	potassium iodide
Drugs	19063297	thyrotropin alfa
Drugs	19069873	sodium iodide

Osteoporosis

KEEPER category	Concept ID	Concept Name

KEEPER category	Concept ID	Concept Name
Disease of Interest	80502	Osteoporosis
Alternative Diagnosis	443392	Malignant neoplastic disease
Comorbidities	75617	Degenerative joint disease involving multiple joints
Comorbidities	77646	Disorder of bone and articular cartilage
Comorbidities	80767	Breast lump
Comorbidities	80816	Degeneration of intervertebral disc
Comorbidities	81611	Diverticular disease of colon
Comorbidities	193739	Ovarian failure
Comorbidities	312648	Benign essential hypertension
Comorbidities	318800	Gastroesophageal reflux disease
Comorbidities	319843	Mitral valve disorder
Comorbidities	374009	Organic mental disorder
Comorbidities	441628	Menopausal and postmenopausal disorders
Comorbidities	4059049	Mammography abnormal
Comorbidities	4113821	Anxiety state
Comorbidities	4172857	Menopause finding
Comorbidities	37017182	Joint pain of pelvic region
Symptoms	74138	Pathological fracture of vertebra
Symptoms	77646	Disorder of bone and articular cartilage
Symptoms	80816	Degeneration of intervertebral disc
Symptoms	434500	Closed fracture of neck of femur
Symptoms	443459	Pathological fracture of neck of femur
Symptoms	4170742	Closed fracture of vertebral column
Symptoms	37017182	Joint pain of pelvic region
Complications	75053	Fracture of bone
Complications	77646	Disorder of bone and articular cartilage
Drugs	1511646	pamidronate
Drugs	1512480	ibandronate
Drugs	1513103	raloxifene
Drugs	1516800	risedronate
Drugs	1524674	zoledronic acid
Drugs	1552929	etidronate
Drugs	1557272	alendronate
Drugs	19011064	tiludronic acid
Drugs	19024249	clodronic acid
Drugs	19115051	risedronic acid
Drugs	40222444	denosumab
Drugs	44506794	bazedoxifene

KEEPER category	Concept ID	Concept Name
Measurements	3009220	Epithelial cells.squamous [#/volume] in Urine sediment
Measurements	4007831	Vitamin D, 25-hydroxy measurement
Measurements	4018619	Screening for occult blood in feces
Measurements	4034850	Laboratory test
Measurements	4062484	Screening for malignant neoplasm of cervix
Measurements	4077697	Screening mammography
Measurements	4114314	Screening for osteoporosis
Measurements	4152047	Hyperlipidemia screening
Measurements	4171430	Screening for malignant neoplasm of rectum
Measurements	4175226	Screening for malignant neoplasm of colon
Measurements	4246879	Thyroid stimulating hormone measurement
Measurements	4262119	Human papillomavirus DNA detection
Diagnostic Procedures	0	No matching concept
Diagnostic Procedures	9201	Inpatient Visit
Diagnostic Procedures	2008419	Other miscellaneous procedures
Diagnostic Procedures	2211359	Radiologic examination, chest; single view, frontal
Diagnostic Procedures	2211361	Radiologic examination, chest, 2 views, frontal and lateral
Diagnostic Procedures	2211439	Radiological supervision and interpretation, percutaneous vertebroplasty, vertebral augmentation, or sacral augmentation (sacroplasty), including cavity creation, per vertebral body or sacrum; under fluoroscopic guidance
Diagnostic Procedures	2211473	Radiologic examination, hip, unilateral; complete, minimum of 2 views
Diagnostic Procedures	2211727	Unlisted diagnostic radiographic procedure
Diagnostic Procedures	2211739	Ultrasound, breast(s) (unilateral or bilateral), real time with image documentation
Diagnostic Procedures	2211808	Computer-aided detection (computer algorithm analysis of digital image data for lesion detection) with further review for interpretation, with or without digitization of film radiographic images; diagnostic mammography (List separately in addition to code
Diagnostic Procedures	2211809	Computer-aided detection (computer algorithm analysis of digital image data for lesion detection) with further review for interpretation, with or without digitization of film radiographic images; screening mammography (List separately in addition to code
Diagnostic Procedures	2211814	Screening mammography, bilateral (2-view study of each breast)
Diagnostic Procedures	2213221	Cytopathology, cervical or vaginal (any reporting system), collected in preservative fluid, automated thin layer preparation; manual screening under physician supervision

KEEPER category	Concept ID	Concept Name
Diagnostic Procedures	2313870	Echocardiography, transthoracic, real-time with image documentation (2D), includes M-mode recording, when performed, complete, without spectral or color Doppler echocardiography
Diagnostic Procedures	4004516	Pre-surgery evaluation
Diagnostic Procedures	4036803	General examination of patient
Diagnostic Procedures	4052851	Transmission bone density study
Diagnostic Procedures	4141759	Long-term drug therapy
Diagnostic Procedures	4159841	Closed reduction of fracture of femur with internal fixation
Diagnostic Procedures	4211987	Open reduction of fracture of femur with internal fixation
Diagnostic Procedures	4297365	Partial hip replacement by prosthesis
Diagnostic Procedures	4306780	Gynecologic examination
Diagnostic Procedures	42737560	Screening mammography, bilateral (two view film study of each breast) (Deprecated)
Diagnostic Procedures	42738270	Blood count; hemogram and platelet count, automated, and automated partial differential WBC count (CBC) (Deprecated)
Diagnostic Procedures	42738308	Unlisted miscellaneous pathology test (Deprecated)
Diagnostic Procedures	42739102	Dual energy x-ray absorptiometry (DEXA), bone density study, one or more sites; axial skeleton (eg, hips, pelvis, spine) (Deprecated)
Diagnostic Procedures	42739103	Dual energy x-ray absorptiometry (DEXA), bone density study, one or more sites; appendicular skeleton (peripheral) (eg, radius, wrist, heel) (Deprecated)
Diagnostic Procedures	42739824	Digitization of film radiographic images with computer analysis for lesion detection and further physician review for interpretation, screening mammography (List separately in addition to code for primary procedure) (Deprecated)
Diagnostic Procedures	42740538	Computer aided detection (computer algorithm analysis of digital image data for lesion detection) with further physician review for interpretation, with or without digitization of film radiographic images; screening mammography (List separ… (Deprecated)
Diagnostic Procedures	42740642	Dual energy x-ray absorptiometry (DXA), bone density study, one or more sites; vertebral fracture assessment (Deprecated)
Diagnostic Procedures	44782057	DRAWING BLOOD FOR SPECIMEN (Deprecated)

Rheumatoid arthritis

KEEPER category	Concept ID	Concept Name

KEEPER category	Concept ID	Concept Name
Disease of Interest	80809	Rheumatoid arthritis
Disease of Interest	2107558	Rheumatoid arthritis (RA) disease activity, low (RA)
Disease of Interest	2107559	Rheumatoid arthritis (RA) disease activity, moderate (RA)
Disease of Interest	2107560	Rheumatoid arthritis (RA) disease activity, high (RA)
Disease of Interest	2107561	Disease prognosis for rheumatoid arthritis assessed, poor prognosis documented (RA)
Disease of Interest	2107572	Disease prognosis for rheumatoid arthritis assessed, good prognosis documented (RA)
Disease of Interest	2108721	Patient receiving first-time biologic disease modifying anti-rheumatic drug therapy for rheumatoid arthritis (RA)
Disease of Interest	4035611	Seropositive rheumatoid arthritis
Disease of Interest	4083556	Seronegative rheumatoid arthritis
Disease of Interest	4102493	Polyneuropathy in rheumatoid arthritis
Disease of Interest	4107913	Myopathy due to rheumatoid arthritis
Disease of Interest	4297650	Cutaneous atrophy due to rheumatoid arthritis
Disease of Interest	4334806	Deformity of foot due to rheumatoid arthritis
Disease of Interest	46273442	Deformity of hand due to rheumatoid arthritis
Alternative Diagnosis	73553	Arthropathy
Alternative Diagnosis	137275	Disorder of muscle
Alternative Diagnosis	140168	Psoriasis
Alternative Diagnosis	255334	Collagen disease
Alternative Diagnosis	434621	Autoimmune disease
Alternative Diagnosis	4143972	Raynaud’s phenomenon
Symptoms	73553	Arthropathy
Symptoms	77074	Joint pain
Symptoms	133299	Swelling of limb
Symptoms	138525	Pain in limb
Symptoms	4013526	ESR raised

KEEPER category	Concept ID	Concept Name
Symptoms	4027396	Peripheral neuritis
Symptoms	4090425	Altered sensation of skin
Comorbidities	255334	Collagen disease
Comorbidities	318800	Gastroesophageal reflux disease
Comorbidities	319843	Mitral valve disorder
Comorbidities	434621	Autoimmune disease
Comorbidities	437264	Tobacco dependence syndrome
Comorbidities	4113821	Anxiety state
Comorbidities	4143972	Raynaud's phenomenon
Comorbidities	4172857	Menopause finding
Drugs	912263	certolizumab pegol
Drugs	937368	infliximab
Drugs	964339	sulfasalazine
Drugs	1119119	adalimumab
Drugs	1151789	etanercept
Drugs	1153664	propoxyphene
Drugs	1305058	methotrexate
Drugs	1777087	hydroxychloroquine
Drugs	19041065	golimumab
Drugs	19111620	folic acid
Measurements	3000620	Complement C3 [Mass/volume] in Serum or Plasma
Measurements	3000905	Leukocytes [#/volume] in Blood by Automated count
Measurements	3000963	Hemoglobin [Mass/volume] in Blood
Measurements	3008898	HLA-B27 [Presence]
Measurements	3009220	Epithelial cells.squamous [#/volume] in Urine sediment
Measurements	3009744	MCHC [Mass/volume] by Automated count
Measurements	3010645	SCL-70 extractable nuclear Ab [Units/volume] in Serum
Measurements	3016520	Beta globulin [Mass/volume] in Serum or Plasma by Electrophoresis
Measurements	3016921	Smith extractable nuclear Ab [Units/volume] in Serum
Measurements	3017766	Complement C4 [Mass/volume] in Serum or Plasma
Measurements	3020416	Erythrocytes [#/volume] in Blood by Automated count
Measurements	3022113	Urinalysis microscopic panel - Urine sediment
Measurements	3023314	Hematocrit [Volume Fraction] of Blood by Automated count
Measurements	3024536	Nuclear Ab pattern [Interpretation] in Serum by Immunofluorescence
Measurements	3027184	Lupus anticoagulant [Interpretation] in Platelet poor plasma
Measurements	3027450	Sjogrens syndrome-B extractable nuclear Ab [Units/volume] in Serum
Measurements	3030367	Cyclic citrullinated peptide IgG Ab [Units/volume] in Serum or Plasma
Measurements	3033159	Nuclear IgG Ab [Units/volume] in Serum by Flow cytometry (FC)
Measurements	3036779	HLA-B27 [Presence] by NAA with probe detection
Measurements	3039162	Centromere protein B Ab [Units/volume] in Serum

KEEPER category	Concept ID	Concept Name
Measurements	3040168	Immature granulocytes [#/volume] in Blood
Measurements	4018467	Measurement of Borrelia burgdorferi antibody
Measurements	4156643	Blood urate measurement
Measurements	4196218	Aldolase measurement
Measurements	4208414	C-reactive protein measurement
Measurements	4234494	Angiotensin converting enzyme measurement
Measurements	4319466	Complete blood count with white cell differential, automated
Measurements	40760212	Cyclic citrullinated peptide IgA+IgG Ab [Units/volume] in Serum or Plasma by Immunoassay
Complications	75897	Polyarthropathy
Complications	77074	Joint pain
Complications	78227	Degenerative joint disease of hand
Complications	138525	Pain in limb
Complications	255334	Collagen disease
Complications	4143972	Raynaud's phenomenon
Complications	4272240	Malaise
Diagnostic Procedures	9201	Inpatient Visit
Diagnostic Procedures	581477	Office Visit
Diagnostic Procedures	2008419	Other miscellaneous procedures
Diagnostic Procedures	2108115	Collection of venous blood by venipuncture
Diagnostic Procedures	2211359	Radiologic examination, chest; single view, frontal
Diagnostic Procedures	2211361	Radiologic examination, chest, 2 views, frontal and lateral
Diagnostic Procedures	2211458	Radiologic examination, hand; 2 views
Diagnostic Procedures	2211459	Radiologic examination, hand; minimum of 3 views
Diagnostic Procedures	2211473	Radiologic examination, hip, unilateral; complete, minimum of 2 views
Diagnostic Procedures	2211490	Radiologic examination, foot; 2 views
Diagnostic Procedures	2211727	Unlisted diagnostic radiographic procedure
Diagnostic Procedures	2212477	Molecular diagnostics; dot/slot blot production, each nucleic acid preparation
Diagnostic Procedures	2313870	Echocardiography, transthoracic, real-time with image documentation (2D), includes M-mode recording, when performed, complete, without spectral or color Doppler echocardiography
Diagnostic	4004516	Pre-surgery evaluation

KEEPER category	Concept ID	Concept Name
Procedures		
Diagnostic Procedures	4141759	Long-term drug therapy
Diagnostic Procedures	4306780	Gynecologic examination
Diagnostic Procedures	42738269	Blood count; hemogram and platelet count, automated, and manual differential WBC count (CBC) (Deprecated)
Diagnostic Procedures	42738270	Blood count; hemogram and platelet count, automated, and automated partial differential WBC count (CBC) (Deprecated)
Diagnostic Procedures	42738308	Unlisted miscellaneous pathology test (Deprecated)
Diagnostic Procedures	44782057	DRAWING BLOOD FOR SPECIMEN (Deprecated)

Viral hepatitis type A

KEEPER category	Concept ID	Concept Name
Disease of Interest	194856	Hepatic coma due to viral hepatitis A
Disease of Interest	4013974	Hepatitis A test positive
Disease of Interest	4223947	Viral hepatitis, type A
Complications	192956	Cholecystitis
Complications	194692	Cirrhosis - non-alcoholic
Complications	194990	Inflammatory disease of liver
Complications	197320	Acute renal failure syndrome
Complications	4029488	Hepatic encephalopathy
Alternative Diagnosis	24966	Esophageal varices
Alternative Diagnosis	137977	Jaundice
Alternative Diagnosis	192240	Chronic viral hepatitis B with hepatitis D
Alternative Diagnosis	192242	Acute hepatitis C
Alternative Diagnosis	193693	Viral hepatitis without hepatic coma
Alternative Diagnosis	194692	Cirrhosis - non-alcoholic
Alternative Diagnosis	194856	Hepatic coma due to viral hepatitis A
Alternative Diagnosis	194990	Inflammatory disease of liver
Alternative	196625	Viral hepatitis A without hepatic coma

KEEPER category	Concept ID	Concept Name
Diagnosis		
Alternative Diagnosis	197493	Hepatitis D superinfection of hepatitis B carrier
Alternative Diagnosis	197494	Viral hepatitis C
Alternative Diagnosis	197795	Acute type B viral hepatitis
Alternative Diagnosis	198683	Viral hepatitis B without hepatic coma
Alternative Diagnosis	198964	Chronic hepatitis C
Alternative Diagnosis	198978	Acute gonorrhea of lower genitourinary tract
Alternative Diagnosis	200031	Hepatic coma due to viral hepatitis B
Alternative Diagnosis	200527	Splenomegaly
Alternative Diagnosis	201343	Acute alcoholic liver disease
Alternative Diagnosis	201613	Chronic nonalcoholic liver disease
Alternative Diagnosis	436238	Elevated level of transaminase and lactic acid dehydrogenase
Alternative Diagnosis	437264	Tobacco dependence syndrome
Alternative Diagnosis	437784	Infectious mononucleosis
Alternative Diagnosis	439672	Hepatic coma due to viral hepatitis C
Alternative Diagnosis	439674	Chronic viral hepatitis B without delta-agent
Alternative Diagnosis	439727	Human immunodeficiency virus infection
Alternative Diagnosis	439926	Malaise and fatigue
Alternative Diagnosis	444367	Cholelithiasis without obstruction
Alternative Diagnosis	763021	Chronic viral hepatitis C with hepatic coma
Alternative Diagnosis	4015842	Viral hepatitis carrier
Alternative Diagnosis	4029488	Hepatic encephalopathy
Alternative Diagnosis	4043371	Inflammatory disorder of digestive tract

KEEPER category	Concept ID	Concept Name
Alternative Diagnosis	4111998	Esophageal varices associated with another disorder
Alternative Diagnosis	4149084	Vaginitis
Alternative Diagnosis	4291005	Viral hepatitis
Alternative Diagnosis	4337543	Hepatic necrosis
Alternative Diagnosis	4340379	Hepatitis B carrier
Alternative Diagnosis	4340380	Hepatitis C carrier
Alternative Diagnosis	40481531	Sequela of chronic liver disease
Alternative Diagnosis	45757141	Viral hepatitis in mother complicating childbirth
Alternative Diagnosis	46270037	Hepatic coma due to acute hepatitis C
Symptoms	27674	Nausea and vomiting
Symptoms	31967	Nausea
Symptoms	137977	Jaundice
Symptoms	200219	Abdominal pain
Symptoms	200528	Ascites
Symptoms	436238	Elevated level of transaminase and lactic acid dehydrogenase
Symptoms	439926	Malaise and fatigue
Symptoms	441408	Vomiting
Symptoms	4047791	Abnormal body temperature
Comorbidities	24966	Esophageal varices
Comorbidities	78818	Large fetus causing disproportion with antenatal problem
Comorbidities	81611	Diverticular disease of colon
Comorbidities	194692	Cirrhosis - non-alcoholic
Comorbidities	194990	Inflammatory disease of liver
Comorbidities	195209	Benign neoplasm of liver and/or biliary ducts
Comorbidities	196170	Liver disorder in pregnancy - delivered
Comorbidities	196436	Internal hemorrhoids
Comorbidities	196463	Alcoholic cirrhosis
Comorbidities	197917	Disorder of biliary tract
Comorbidities	198978	Acute gonorrhea of lower genitourinary tract
Comorbidities	200527	Splenomegaly
Comorbidities	200528	Ascites
Comorbidities	201072	Benign prostatic hyperplasia without outflow obstruction
Comorbidities	201343	Acute alcoholic liver disease

KEEPER category	Concept ID	Concept Name
Comorbidities	201613	Chronic nonalcoholic liver disease
Comorbidities	253954	Pulmonary tuberculosis
Comorbidities	318800	Gastroesophageal reflux disease
Comorbidities	437264	Tobacco dependence syndrome
Comorbidities	437784	Infectious mononucleosis
Comorbidities	439727	Human immunodeficiency virus infection
Comorbidities	443410	Infective pneumonia
Comorbidities	4015842	Viral hepatitis carrier
Comorbidities	4029488	Hepatic encephalopathy
Comorbidities	4043371	Inflammatory disorder of digestive tract
Comorbidities	4113821	Anxiety state
Comorbidities	4151863	Congenital abnormality of liver and/or biliary tract
Comorbidities	4273323	Rubella
Comorbidities	4337543	Hepatic necrosis
Comorbidities	40481531	Sequela of chronic liver disease
Drugs	1714165	peginterferon alfa-2a
Drugs	1762711	ribavirin
Drugs	1797155	peginterferon alfa-2b
Measurements	2213126	Infectious agent detection by nucleic acid (DNA or RNA); hepatitis B virus, amplified probe technique
Measurements	2213127	Infectious agent detection by nucleic acid (DNA or RNA); hepatitis B virus, quantification
Measurements	2213130	Infectious agent detection by nucleic acid (DNA or RNA); hepatitis C, quantification, includes reverse transcription when performed
Measurements	2213142	Infectious agent detection by nucleic acid (DNA or RNA); HIV-1, quantification, includes reverse transcription when performed
Measurements	3000905	Leukocytes [#/volume] in Blood by Automated count
Measurements	3000963	Hemoglobin [Mass/volume] in Blood
Measurements	3003215	Lymphocytes [#/volume] in Blood by Manual count
Measurements	3003470	Hepatitis C virus Ab [Units/volume] in Serum by Immunoassay
Measurements	3003974	HIV 1 IgG Ab [Presence] in Serum
Measurements	3006453	Hepatitis B virus surface Ab [Presence] in Serum by Immunoassay
Measurements	3006923	Alanine aminotransferase [Enzymatic activity/volume] in Serum or Plasma
Measurements	3007359	Bilirubin.indirect [Mass/volume] in Serum or Plasma
Measurements	3009220	Epithelial cells.squamous [#/volume] in Urine sediment
Measurements	3009744	MCHC [Mass/volume] by Automated count
Measurements	3010229	Hepatitis B virus core IgM Ab [Units/volume] in Serum or Plasma by Immunoassay
Measurements	3011516	Hepatitis B virus e Ag [Units/volume] in Serum by Immunoassay
Measurements	3012030	MCH [Entitic mass] by Automated count

KEEPER category	Concept ID	Concept Name
Measurements	3013721	Aspartate aminotransferase [Enzymatic activity/volume] in Serum or Plasma
Measurements	3016557	Hepatitis B virus core Ab [Units/volume] in Serum by Immunoassay
Measurements	3018447	Hepatitis C virus RNA [Units/volume] (viral load) in Serum or Plasma by NAA with probe detection
Measurements	3018867	Mumps virus IgG Ab [Units/volume] in Serum
Measurements	3019510	Hepatitis B virus surface Ag [Presence] in Serum or Plasma by Immunoassay
Measurements	3019676	Bilirubin.conjugated [Mass/volume] in Serum or Plasma
Measurements	3020029	Reducing substances [Presence] in Urine
Measurements	3020316	Hepatitis A virus IgM Ab [Presence] in Serum or Plasma by Immunoassay
Measurements	3020416	Erythrocytes [#/volume] in Blood by Automated count
Measurements	3020509	Albumin/Globulin [Mass Ratio] in Serum or Plasma
Measurements	3020630	Protein [Mass/volume] in Serum or Plasma
Measurements	3022113	Urinalysis microscopic panel - Urine sediment
Measurements	3022560	Hepatitis B virus core IgM Ab [Presence] in Serum or Plasma by Immunoassay
Measurements	3023314	Hematocrit [Volume Fraction] of Blood by Automated count
Measurements	3023378	Hepatitis B virus e Ag [Presence] in Serum or Plasma by Immunoassay
Measurements	3023599	MCV [Entitic volume] by Automated count
Measurements	3023884	Cholesterol in HDL/Cholesterol.total [Mass Ratio] in Serum or Plasma
Measurements	3024128	Bilirubin.total [Mass/volume] in Serum or Plasma
Measurements	3024557	Polymorphonuclear cells/100 leukocytes in Blood by Manual count
Measurements	3024561	Albumin [Mass/volume] in Serum or Plasma
Measurements	3024929	Platelets [#/volume] in Blood by Automated count
Measurements	3027245	Hepatitis A virus IgM Ab [Units/volume] in Serum by Immunoassay
Measurements	3027346	Hepatitis B virus DNA [#/volume] (viral load) in Serum or Plasma by NAA with probe detection
Measurements	3028089	Alkaline phosphatase isoenzyme [Units/volume] in Serum or Plasma
Measurements	3028662	Hepatitis A virus Ab [Units/volume] in Serum or Plasma by Immunoassay
Measurements	3031004	Mononuclear cells/100 leukocytes in Blood by Manual count
Measurements	3031806	HLA-B*57:01 [Presence] in Specimen
Measurements	3034868	Hepatitis C virus RNA [log units/volume] (viral load) in Serum or Plasma by NAA with probe detection
Measurements	3035456	Hepatitis A virus Ab [Presence] in Serum by Immunoassay
Measurements	3035962	HIV 1+2 Ab [Presence] in Serum or Plasma by Immunoassay
Measurements	3035995	Alkaline phosphatase [Enzymatic activity/volume] in Serum or Plasma
Measurements	3036282	Hepatitis B virus core Ab [Presence] in Serum or Plasma by Immunoassay
Measurements	3039218	Hepatitis A virus Ab [Interpretation] in Serum
Measurements	3049147	HIV 1+O+2 Ab [Units/volume] in Serum or Plasma
Measurements	3053246	HIV 1+O+2 Ab [Presence] in Serum or Plasma

KEEPER category	Concept ID	Concept Name
Measurements	4014768	Alpha-1-fetoprotein measurement, serum
Measurements	4034850	Laboratory test
Measurements	4062484	Screening for malignant neoplasm of cervix
Measurements	4064905	Venereal disease screening
Measurements	4098519	LDH blood measurement
Measurements	4175226	Screening for malignant neoplasm of colon
Measurements	4216632	Bilirubin, direct measurement
Measurements	4245982	Ammonia measurement
Measurements	4276028	Amylase measurement
Measurements	4289475	Gamma glutamyl transferase measurement
Measurements	4319466	Complete blood count with white cell differential, automated
Measurements	4324147	Alpha-1-antitrypsin measurement
Measurements	4337961	Ceruloplasmin measurement
Diagnostic Procedures	0	No matching concept
Diagnostic Procedures	9201	Inpatient Visit
Diagnostic Procedures	581477	Office Visit
Diagnostic Procedures	2002449	Endoscopic excision or destruction of lesion or tissue of esophagus
Diagnostic Procedures	2003287	Endoscopic sphincterotomy and papillotomy
Diagnostic Procedures	2006932	Diagnostic ultrasound of abdomen and retroperitoneum
Diagnostic Procedures	2008008	Continuous invasive mechanical ventilation for less than 96 consecutive hours
Diagnostic Procedures	2008419	Other miscellaneous procedures
Diagnostic Procedures	2100978	Anesthesia for lower intestinal endoscopic procedures, endoscope introduced distal to duodenum
Diagnostic Procedures	2108329	Transcatheter biopsy
Diagnostic Procedures	2211359	Radiologic examination, chest; single view, frontal
Diagnostic Procedures	2211361	Radiologic examination, chest, 2 views, frontal and lateral
Diagnostic Procedures	2211424	Computed tomography, pelvis; with contrast material(s)
Diagnostic Procedures	2211506	Radiologic examination, abdomen; single anteroposterior view
Diagnostic Procedures	2211511	Computed tomography, abdomen; with contrast material(s)
Diagnostic	2211512	Computed tomography, abdomen; without contrast material, followed by

KEEPER category	Concept ID	Concept Name
Procedures		contrast material(s) and further sections
Diagnostic Procedures	2211655	Transcatheter biopsy, radiological supervision and interpretation
Diagnostic Procedures	2211727	Unlisted diagnostic radiographic procedure
Diagnostic Procedures	2211740	Ultrasound, abdominal, real time with image documentation; complete
Diagnostic Procedures	2211741	Ultrasound, abdominal, real time with image documentation; limited (eg, single organ, quadrant, follow-up)
Diagnostic Procedures	2213418	Immunization administration (includes percutaneous, intradermal, subcutaneous, or intramuscular injections); 1 vaccine (single or combination vaccine/toxoid)
Diagnostic Procedures	2313870	Echocardiography, transthoracic, real-time with image documentation (2D), includes M-mode recording, when performed, complete, without spectral or color Doppler echocardiography
Diagnostic Procedures	4004516	Pre-surgery evaluation
Diagnostic Procedures	4013354	Insertion of endotracheal tube
Diagnostic Procedures	4036803	General examination of patient
Diagnostic Procedures	4119971	Vascular cannula adjustment
Diagnostic Procedures	4120443	Bone marrow sampling
Diagnostic Procedures	4123999	Percutaneous needle biopsy of liver
Diagnostic Procedures	4166920	Endoscopic biopsy of rectum
Diagnostic Procedures	4167261	Bronchoscopy and biopsy of bronchus
Diagnostic Procedures	4216752	Drug therapy
Diagnostic Procedures	4231419	Abdominal paracentesis
Diagnostic Procedures	4286821	Anesthesia for upper gastrointestinal endoscopic procedure
Diagnostic Procedures	4289454	Venous catheterization for renal dialysis
Diagnostic Procedures	4306780	Gynecologic examination
Diagnostic Procedures	42738267	Blood count; hemogram, automated (RBC, WBC, Hgb, Hct and indices only) (Deprecated)
Diagnostic Procedures	42738268	Blood count; hemogram, automated, and manual differential WBC count (CBC) (Deprecated)
Diagnostic	42738269	Blood count; hemogram and platelet count, automated, and manual

KEEPER category	Concept ID	Concept Name
Procedures		differential WBC count (CBC) (Deprecated)
Diagnostic Procedures	42738270	Blood count; hemogram and platelet count, automated, and automated partial differential WBC count (CBC) (Deprecated)
Diagnostic Procedures	42738308	Unlisted miscellaneous pathology test (Deprecated)
Diagnostic Procedures	42739548	Sedation with or without analgesia (conscious sedation); intravenous, intramuscular or inhalation (Deprecated)
Diagnostic Procedures	42740674	Immunization administration younger than 8 years of age (includes percutaneous, intradermal, subcutaneous, or intramuscular injections) when the physician counsels the patient/family; first injection (single or combination vaccine/toxoid), per day
Diagnostic Procedures	44782057	DRAWING BLOOD FOR SPECIMEN (Deprecated)

Diabetes mellitus type I

KEEPER category	Concept ID	Concept Name
Disease of Interest	201254	Type 1 diabetes mellitus
Disease of Interest	435216	Disorder due to type 1 diabetes mellitus
Symptoms	0	
Comorbidities	192963	Disorder of pancreas
Comorbidities	201820	Diabetes mellitus
Comorbidities	320128	Essential hypertension
Comorbidities	375545	Cataract
Comorbidities	380834	Coma
Comorbidities	433736	Obesity
Comorbidities	433968	Candidiasis
Comorbidities	441267	Cystic fibrosis
Comorbidities	442793	Complication due to diabetes mellitus
Comorbidities	443238	Diabetic - poor control
Comorbidities	4016045	Diabetic - good control
Comorbidities	4034964	Abnormal metabolic state due to diabetes mellitus
Comorbidities	4051114	Family history of diabetes mellitus
Comorbidities	4065354	Neuropathic ulcer of foot due to diabetes mellitus
Comorbidities	4159742	Diabetic foot ulcer
Comorbidities	4163735	Hemochromatosis
Comorbidities	4170226	Disorder of lipoprotein AND/OR lipid metabolism
Comorbidities	4209145	Ketoacidosis
Comorbidities	4226354	Gangrene due to diabetes mellitus
Comorbidities	4299544	Acanthosis nigricans
Comorbidities	40443308	Polycystic ovary syndrome
Comorbidities	43530690	Foot ulcer due to type 2 diabetes mellitus
Comorbidities	45757392	Hypertension concurrent and due to end stage renal disease on dialysis due to type 2 diabetes mellitus

KEEPER category	Concept ID	Concept Name
Drugs	741530	orlistat
Drugs	1502809	tolazamide
Drugs	1502826	nateglinide
Drugs	1502905	insulin glargine
Drugs	1503297	metformin
Drugs	1510202	miglitol
Drugs	1513876	insulin lispro protamine, human
Drugs	1515249	troglitazone
Drugs	1516766	repaglinide
Drugs	1516976	insulin detemir
Drugs	1517998	pramlintide
Drugs	1525215	pioglitazone
Drugs	1529331	acarbose
Drugs	1530014	acetohexamide
Drugs	1531601	insulin aspart protamine, human
Drugs	1544838	insulin glulisine, human
Drugs	1547504	rosiglitazone
Drugs	1550023	insulin lispro
Drugs	1559684	glyburide
Drugs	1560171	glipizide
Drugs	1567198	insulin aspart, human
Drugs	1580747	sitagliptin
Drugs	1583722	exenatide
Drugs	1594973	chlorpropamide
Drugs	1597756	glimepiride
Drugs	19067100	Glucotrol XL
Drugs	19122121	Insulin
Drugs	21600713	INSULINS AND ANALOGUES
Drugs	42873378	phentermine / topiramate Extended Release Oral Capsule [Qsymia]
Drugs	45774489	bupropion / naltrexone Extended Release Oral Tablet [Contrave]
Diagnostic Procedures	0	
Measurements	3005131	Glucose mean value [Mass/volume] in Blood Estimated from glycated hemoglobin
Measurements	3005446	Hemoglobin A1/Hemoglobin.total in Blood
Measurements	3005673	Hemoglobin A1c/Hemoglobin.total in Blood by HPLC
Measurements	3033145	Hemoglobin A1c measurement device panel
Measurements	3033819	Glucose-6-Phosphate dehydrogenase [Presence] in Serum
Measurements	4018317	Glucose measurement, quantitative
Measurements	4035259	Anti-pancreatic islet cell antibody
Measurements	4041697	Blood glucose method

KEEPER category	Concept ID	Concept Name
Measurements	4144235	Glucose measurement, blood
Measurements	4149883	Glucose measurement, urine
Measurements	4184637	Hemoglobin A1c measurement
Measurements	4290342	Insulin antibody measurement
Measurements	37021662	C peptide | Serum or Plasma | Chemistry - non-challenge
Measurements	37393411	Islet cell antibody level
Measurements	40652732	C peptide|Mass Concentration|Moment in time|Blood, Serum or Plasma
Measurements	40653487	Pancreatic islet cell Ab|Presence or Threshold|Moment in time|Blood, Serum or Plasma
Measurements	40654476	C peptide|Moment in time|Serum or Plasma
Measurements	40759806	Pancreatic islet cell IgG Ab [Titer] in Serum by Immunofluorescence
Measurements	40785865	C peptide
Alternative Diagnosis	192963	Disorder of pancreas
Alternative Diagnosis	201820	Diabetes mellitus
Alternative Diagnosis	201826	Type 2 diabetes mellitus
Alternative Diagnosis	320128	Essential hypertension
Alternative Diagnosis	375545	Cataract
Alternative Diagnosis	380834	Coma
Alternative Diagnosis	433736	Obesity
Alternative Diagnosis	433968	Candidiasis
Alternative Diagnosis	441267	Cystic fibrosis
Alternative Diagnosis	442793	Complication due to diabetes mellitus
Alternative Diagnosis	443238	Diabetic - poor control
Alternative Diagnosis	4016045	Diabetic - good control
Alternative Diagnosis	4034964	Abnormal metabolic state due to diabetes mellitus
Alternative Diagnosis	4051114	Family history of diabetes mellitus
Alternative Diagnosis	4065354	Neuropathic ulcer of foot due to diabetes mellitus
Alternative Diagnosis	4159742	Diabetic foot ulcer
Alternative	4163735	Hemochromatosis

KEEPER category	Concept ID	Concept Name
Diagnosis		
Alternative Diagnosis	4170226	Disorder of lipoprotein AND/OR lipid metabolism
Alternative Diagnosis	4209145	Ketoacidosis
Alternative Diagnosis	4226354	Gangrene due to diabetes mellitus
Alternative Diagnosis	4299544	Acanthosis nigricans
Alternative Diagnosis	40443308	Polycystic ovary syndrome
Alternative Diagnosis	43530690	Foot ulcer due to type 2 diabetes mellitus
Alternative Diagnosis	45757392	Hypertension concurrent and due to end stage renal disease on dialysis due to type 2 diabetes mellitus
Treatment Procedures	2746766	Excision of Stomach, Percutaneous Endoscopic Approach, Vertical
Treatment Procedures	2746768	Excision of Stomach, Percutaneous Endoscopic Approach
Treatment Procedures	4002199	Pancreas implantation
Treatment Procedures	4143852	Laparoscopic sleeve gastrectomy
Treatment Procedures	40756884	Laparoscopy, surgical, gastric restrictive procedure; longitudinal gastrectomy (ie, sleeve gastrectomy)
Complications	442793	Complication due to diabetes mellitus

Acute appendicitis

KEEPER category	Concept ID	Concept Name
Disease of Interest	440448	Appendicitis
Symptoms	0	
Comorbidities	0	
Drugs	997881	clindamycin
Drugs	1702364	ticarcillin
Drugs	1707164	metronidazole
Drugs	1709170	meropenem
Drugs	1717327	ampicillin
Drugs	1717963	ertapenem
Drugs	1741122	tazobactam
Drugs	1742253	levofloxacin
Drugs	1746114	piperacillin
Drugs	1748975	cefepime
Drugs	1759842	clavulanate

KEEPER category	Concept ID	Concept Name
Drugs	1836241	sulbactam
Drugs	45892419	gentamicin
Diagnostic Procedures	724944	Radiologic examination, abdomen; 1 view
Diagnostic Procedures	724945	Radiologic examination, abdomen; 2 views
Diagnostic Procedures	724988	Radiologic examination, abdomen; 3 or more views
Diagnostic Procedures	725003	Anesthesia for upper gastrointestinal endoscopic procedures, endoscope introduced proximal to duodenum; not otherwise specified
Diagnostic Procedures	725004	Anesthesia for upper gastrointestinal endoscopic procedures, endoscope introduced proximal to duodenum; endoscopic retrograde cholangiopancreatography (ERCP)
Diagnostic Procedures	725005	Anesthesia for lower intestinal endoscopic procedures, endoscope introduced distal to duodenum; not otherwise specified
Diagnostic Procedures	725007	Anesthesia for combined upper and lower gastrointestinal endoscopic procedures, endoscope introduced both proximal to and distal to the duodenum
Diagnostic Procedures	725068	Radiologic examination, chest; single view
Diagnostic Procedures	725069	Radiologic examination, chest; 2 views
Diagnostic Procedures	2002442	Other esophagoscopy
Diagnostic Procedures	2002685	Other endoscopy of small intestine
Diagnostic Procedures	2002946	Open biopsy of rectum
Diagnostic Procedures	2003446	Other laparoscopic umbilical herniorrhaphy
Diagnostic Procedures	2003447	Other open umbilical herniorrhaphy
Diagnostic Procedures	2003502	Other laparotomy
Diagnostic Procedures	2003507	Closed [percutaneous] [needle] biopsy of intra-abdominal mass
Diagnostic Procedures	2003510	Excision or destruction of lesion or tissue of abdominal wall or umbilicus
Diagnostic Procedures	2003511	Excision or destruction of peritoneal tissue
Diagnostic Procedures	2003524	Other lysis of peritoneal adhesions
Diagnostic Procedures	2003783	Other cystoscopy
Diagnostic	2006932	Diagnostic ultrasound of abdomen and retroperitoneum

KEEPER category	Concept ID	Concept Name
Procedures		
Diagnostic Procedures	2006935	Other diagnostic ultrasound
Diagnostic Procedures	2100901	Anesthesia for procedures in lumbar region; diagnostic or therapeutic lumbar puncture
Diagnostic Procedures	2100921	Anesthesia for procedures on upper anterior abdominal wall; not otherwise specified
Diagnostic Procedures	2100938	Anesthesia for upper gastrointestinal endoscopic procedures, endoscope introduced proximal to duodenum
Diagnostic Procedures	2108883	Esophagogastroduodenoscopy, flexible, transoral; diagnostic, including collection of specimen(s) by brushing or washing, when performed (separate procedure)
Diagnostic Procedures	2108886	Esophagogastroduodenoscopy, flexible, transoral; with transendoscopic ultrasound-guided intramural or transmural fine needle aspiration/biopsy(s), (includes endoscopic ultrasound examination limited to the esophagus, stomach or duodenum, and adjacent stru
Diagnostic Procedures	2108887	Esophagogastroduodenoscopy, flexible, transoral; with biopsy, single or multiple
Diagnostic Procedures	2108888	Esophagogastroduodenoscopy, flexible, transoral; with transmural drainage of pseudocyst (includes placement of transmural drainage catheter[s]/stent[s], when performed, and endoscopic ultrasound, when performed)
Diagnostic Procedures	2108890	Esophagogastroduodenoscopy, flexible, transoral; with transendoscopic ultrasound-guided intramural or transmural fine needle aspiration/biopsy(s) (includes endoscopic ultrasound examination of the esophagus, stomach, and either the duodenum or a surgicall
Diagnostic Procedures	2108904	Esophagogastroduodenoscopy, flexible, transoral; with endoscopic ultrasound examination, including the esophagus, stomach, and either the duodenum or a surgically altered stomach where the jejunum is examined distal to the anastomosis
Diagnostic Procedures	2109089	Small intestinal endoscopy, enteroscopy beyond second portion of duodenum, not including ileum; with biopsy, single or multiple
Diagnostic Procedures	2109103	Ileoscopy, through stoma; with biopsy, single or multiple
Diagnostic Procedures	2109180	Sigmoidoscopy, flexible; diagnostic, including collection of specimen(s) by brushing or washing, when performed (separate procedure)
Diagnostic Procedures	2109181	Sigmoidoscopy, flexible; with biopsy, single or multiple
Diagnostic Procedures	2109194	Colonoscopy, flexible; diagnostic, including collection of specimen(s) by brushing or washing, when performed (separate procedure)
Diagnostic Procedures	2109201	Colonoscopy, flexible; with removal of tumor(s), polyp(s), or other lesion(s) by snare technique
Diagnostic Procedures	2109310	Biopsy of liver, needle; when done for indicated purpose at time of other major procedure (List separately in addition to code for primary procedure)
Diagnostic Procedures	2109465	Exchange of previously placed abscess or cyst drainage catheter under radiological guidance (separate procedure)

KEEPER category	Concept ID	Concept Name
Diagnostic Procedures	2109567	Renal biopsy; by surgical exposure of kidney
Diagnostic Procedures	2109586	Renal allotransplantation, implantation of graft; without recipient nephrectomy
Diagnostic Procedures	2109766	Cystourethroscopy (separate procedure)
Diagnostic Procedures	2211426	Magnetic resonance (eg, proton) imaging, pelvis; without contrast material(s)
Diagnostic Procedures	2211427	Magnetic resonance (eg, proton) imaging, pelvis; with contrast material(s)
Diagnostic Procedures	2211428	Magnetic resonance (eg, proton) imaging, pelvis; without contrast material(s), followed by contrast material(s) and further sequences
Diagnostic Procedures	2211493	Radiologic examination; toe(s), minimum of 2 views
Diagnostic Procedures	2211514	Magnetic resonance (eg, proton) imaging, abdomen; without contrast material(s)
Diagnostic Procedures	2211515	Magnetic resonance (eg, proton) imaging, abdomen; with contrast material(s)
Diagnostic Procedures	2211516	Magnetic resonance (eg, proton) imaging, abdomen; without contrast material(s), followed by with contrast material(s) and further sequences
Diagnostic Procedures	2211639	Percutaneous placement of IVC filter, radiological supervision and interpretation
Diagnostic Procedures	2211740	Ultrasound, abdominal, real time with image documentation; complete
Diagnostic Procedures	2211741	Ultrasound, abdominal, real time with image documentation; limited (eg, single organ, quadrant, follow-up)
Diagnostic Procedures	2211742	Ultrasound, retroperitoneal (eg, renal, aorta, nodes), real time with image documentation; complete
Diagnostic Procedures	2211743	Ultrasound, retroperitoneal (eg, renal, aorta, nodes), real time with image documentation; limited
Diagnostic Procedures	2211744	Ultrasound, transplanted kidney, real time and duplex Doppler with image documentation
Diagnostic Procedures	2211768	Ultrasound, pelvic (nonobstetric), real time with image documentation; limited or follow-up (eg, for follicles)
Diagnostic Procedures	2211769	Ultrasound, scrotum and contents
Diagnostic Procedures	2211950	Hepatobiliary ductal system imaging, including gallbladder, with or without pharmacologic intervention, with or without quantitative measurement of gallbladder function
Diagnostic Procedures	2313699	Binocular microscopy (separate diagnostic procedure)
Diagnostic Procedures	2313828	External electrocardiographic recording up to 48 hours by continuous rhythm recording and storage; includes recording, scanning analysis with report, review and interpretation by a physician or other qualified health care professional
Diagnostic	2313992	Duplex scan of arterial inflow and venous outflow of abdominal, pelvic,

KEEPER category	Concept ID	Concept Name
Procedures		scrotal contents and/or retroperitoneal organs; complete study
Diagnostic Procedures	2313993	Duplex scan of arterial inflow and venous outflow of abdominal, pelvic, scrotal contents and/or retroperitoneal organs; limited study
Diagnostic Procedures	2722221	Hysteroscopy, diagnostic (separate procedure). (Deprecated)
Diagnostic Procedures	2722222	Hysteroscopy, surgical; with sampling (biopsy) of endometrium and/or polypectomy, with or without D & C. (Deprecated)
Diagnostic Procedures	2746590	Release Small Intestine, Open Approach
Diagnostic Procedures	2746607	Release Ileum, Percutaneous Endoscopic Approach
Diagnostic Procedures	2746773	Excision of Stomach, Via Natural or Artificial Opening Endoscopic, Diagnostic
Diagnostic Procedures	2746810	Release Peritoneum, Open Approach
Diagnostic Procedures	2747541	Excision of Lesser Omentum, Percutaneous Endoscopic Approach, Diagnostic (Deprecated)
Diagnostic Procedures	2747549	Excision of Peritoneum, Open Approach, Diagnostic
Diagnostic Procedures	2773682	Excision of Right Kidney, Percutaneous Approach, Diagnostic
Diagnostic Procedures	2773692	Excision of Left Kidney, Percutaneous Approach, Diagnostic
Diagnostic Procedures	2776180	Bypass Bilateral Ureters to Ileocutaneous, Open Approach
Diagnostic Procedures	2779576	Drainage of Peritoneal Cavity, Percutaneous Endoscopic Approach, Diagnostic
Diagnostic Procedures	2793091	Magnetic Resonance Imaging (MRI) of Abdomen using Other Contrast
Diagnostic Procedures	2793092	Magnetic Resonance Imaging (MRI) of Abdomen
Diagnostic Procedures	4045438	Rigid proctosigmoidoscopy
Diagnostic Procedures	4052532	Hysteroscopy
Diagnostic Procedures	4085764	Percutaneous aspiration of liver
Diagnostic Procedures	4123999	Percutaneous needle biopsy of liver
Diagnostic Procedures	4167416	Renal lithotripsy
Diagnostic Procedures	4175226	Screening for malignant neoplasm of colon
Diagnostic Procedures	4178367	Radiographic imaging procedure

KEEPER category	Concept ID	Concept Name
Diagnostic Procedures	4207654	Diagnostic ultrasound of gravid uterus
Diagnostic Procedures	4218549	Small intestinal endoscopy with biopsy
Diagnostic Procedures	4220239	Open biopsy of liver
Diagnostic Procedures	4230660	Anoscopy
Diagnostic Procedures	4231419	Abdominal paracentesis
Diagnostic Procedures	4241100	Reduction mammoplasty
Diagnostic Procedures	4249160	Open biopsy of large intestine
Diagnostic Procedures	4249893	Colonoscopy
Diagnostic Procedures	4251314	Laparoscopy
Diagnostic Procedures	4253523	Exploratory laparotomy
Diagnostic Procedures	42742552	Hepatobiliary system imaging, including gallbladder when present
Diagnostic Procedures	43527935	Esophagogastroduodenoscopy, flexible, transoral; with optical endomicroscopy
Diagnostic Procedures	46257516	Colonoscopy, flexible; with endoscopic mucosal resection
Diagnostic Procedures	46273536	Laparoscopic biopsy of ovary
Measurements	3000905	Leukocytes [#/volume] in Blood by Automated count
Measurements	3013650	Neutrophils [#/volume] in Blood by Automated count
Measurements	3017732	Neutrophils [#/volume] in Blood
Measurements	3018010	Neutrophils/100 leukocytes in Blood
Alternative Diagnosis	201618	Disorder of intestine
Alternative Diagnosis	444089	Disorder of abdomen
Alternative Diagnosis	4171379	Disorder of the genitourinary system
Alternative Diagnosis	43531054	Disorder of pelvis
Treatment Procedures	2000219	Other peripheral nerve or ganglion decompression or lysis of adhesions
Treatment Procedures	2000811	Laparoscopic cecectomy
Treatment	2001423	Excision of lesion or tissue of diaphragm

KEEPER category	Concept ID	Concept Name
Procedures		
Treatment Procedures	2002531	Other gastrostomy
Treatment Procedures	2002724	Local excision of lesion or tissue of small intestine, except duodenum
Treatment Procedures	2002747	Other partial resection of small intestine
Treatment Procedures	2002751	Open and other cecectomy
Treatment Procedures	2002762	Open and other right hemicolectomy
Treatment Procedures	2002785	Other small-to-large intestinal anastomosis
Treatment Procedures	2002869	Suture of laceration of large intestine
Treatment Procedures	2002909	Other appendectomy
Treatment Procedures	2002911	Laparoscopic incidental appendectomy
Treatment Procedures	2002922	Other incidental appendectomy
Treatment Procedures	2002964	Local excision of rectal lesion or tissue
Treatment Procedures	2003510	Excision or destruction of lesion or tissue of abdominal wall or umbilicus
Treatment Procedures	2003511	Excision or destruction of peritoneal tissue
Treatment Procedures	2003565	Other operations of abdominal region
Treatment Procedures	2003626	Other kidney transplantation
Treatment Procedures	2003898	Other lysis of perirenal or periureteral adhesions
Treatment Procedures	2004230	Other local excision or destruction of ovary
Treatment Procedures	2004269	Other reimplantation of ovary
Treatment Procedures	2004449	Other excision or destruction of lesion of uterus
Treatment Procedures	2004491	Other dilation and curettage
Treatment Procedures	2004503	Other excision or destruction of uterus and supporting structures
Treatment Procedures	2004643	Other operations on cul-de-sac

KEEPER category	Concept ID	Concept Name
Treatment Procedures	2004788	Removal of extratubal ectopic pregnancy
Treatment Procedures	2008268	Injection of other anti-infective
Treatment Procedures	2100941	Anesthesia for hernia repairs in upper abdomen; lumbar and ventral (incisional) hernias and/or wound dehiscence
Treatment Procedures	2100992	Anesthesia for hernia repairs in lower abdomen; not otherwise specified
Treatment Procedures	2101056	Anesthesia for; anorectal procedure
Treatment Procedures	2101807	Anesthesia for cesarean delivery only
Treatment Procedures	2101813	Neuraxial labor analgesia/anesthesia for planned vaginal delivery (this includes any repeat subarachnoid needle placement and drug injection and/or any necessary replacement of an epidural catheter during labor)
Treatment Procedures	2101877	Incision and drainage of abscess (eg, carbuncle, suppurative hidradenitis, cutaneous or subcutaneous abscess, cyst, furuncle, or paronychia); simple or single
Treatment Procedures	2101888	Puncture aspiration of abscess, hematoma, bulla, or cyst
Treatment Procedures	2108476	Laparoscopy, surgical; with retroperitoneal lymph node sampling (biopsy), single or multiple
Treatment Procedures	2109017	Enterolysis (freeing of intestinal adhesion) (separate procedure)
Treatment Procedures	2109024	Correction of malrotation by lysis of duodenal bands and/or reduction of midgut volvulus (eg, Ladd procedure)
Treatment Procedures	2109028	Enterectomy, resection of small intestine; single resection and anastomosis
Treatment Procedures	2109040	Mobilization (take-down) of splenic flexure performed in conjunction with partial colectomy (List separately in addition to primary procedure)
Treatment Procedures	2109041	Colectomy, partial; with anastomosis
Treatment Procedures	2109056	Colectomy, partial, with removal of terminal ileum with ileocolostomy
Treatment Procedures	2109063	Laparoscopy, surgical; enterectomy, resection of small intestine, single resection and anastomosis
Treatment Procedures	2109066	Laparoscopy, surgical; colectomy, partial, with removal of terminal ileum with ileocolostomy
Treatment Procedures	2109116	Suture of small intestine (enterorrhaphy) for perforated ulcer, diverticulum, wound, injury or rupture; single perforation
Treatment Procedures	2109139	Incision and drainage of appendiceal abscess, open
Treatment Procedures	2109140	Incision and drainage of appendiceal abscess; percutaneous
Treatment Procedures	2109141	Appendectomy

KEEPER category	Concept ID	Concept Name
Treatment Procedures	2109142	Appendectomy; when done for indicated purpose at time of other major procedure (not as separate procedure) (List separately in addition to code for primary procedure)
Treatment Procedures	2109143	Appendectomy; for ruptured appendix with abscess or generalized peritonitis
Treatment Procedures	2109144	Laparoscopy, surgical, appendectomy
Treatment Procedures	2109145	Unlisted laparoscopy procedure, appendix
Treatment Procedures	2109146	Transrectal drainage of pelvic abscess
Treatment Procedures	2109312	Hepatotomy; for percutaneous drainage of abscess or cyst, 1 or 2 stages
Treatment Procedures	2109366	Laparoscopy, surgical; cholecystoenterostomy
Treatment Procedures	2109432	Drainage of peritoneal abscess or localized peritonitis, exclusive of appendiceal abscess; percutaneous
Treatment Procedures	2109435	Drainage of retroperitoneal abscess, open
Treatment Procedures	2109444	Excision or destruction, open, intra-abdominal tumors, cysts or endometriomas, 1 or more peritoneal, mesenteric, or retroperitoneal primary or secondary tumors; largest tumor 5 cm diameter or less
Treatment Procedures	2109453	Laparoscopy, surgical; with aspiration of cavity or cyst (eg, ovarian cyst) (single or multiple)
Treatment Procedures	2109669	Continent diversion, including intestine anastomosis using any segment of small and/or large intestine (Kock pouch or Camey enterocystoplasty)
Treatment Procedures	2109701	Cystostomy, cystotomy with drainage
Treatment Procedures	2109748	Cystoplasty or cystourethroplasty, plastic operation on bladder and/or vesical neck (anterior Y-plasty, vesical fundus resection), any procedure, with or without wedge resection of posterior vesical neck
Treatment Procedures	2110001	Excision of hydrocele; unilateral
Treatment Procedures	2110239	Laparoscopy, surgical; with lysis of adhesions (salpingolysis, ovariolysis) (separate procedure)
Treatment Procedures	2110257	Drainage of ovarian abscess; abdominal approach
Treatment Procedures	2110258	Drainage of pelvic abscess, transvaginal or transrectal approach, percutaneous (eg, ovarian, pericolic)
Treatment Procedures	2110308	Vaginal delivery only (with or without episiotomy and/or forceps)
Treatment Procedures	2110316	Cesarean delivery only
Treatment Procedures	2110330	Induced abortion, by dilation and evacuation
Treatment	2110394	Laparoscopy, surgical, with adrenalectomy, partial or complete, or

KEEPER category	Concept ID	Concept Name
Procedures		exploration of adrenal gland with or without biopsy, transabdominal, lumbar or dorsal
Treatment Procedures	2722201	Laparoscopy, surgical; with fulguration or excision of lesions of the ovary, pelvic viscera, or peritoneal surface by any method. (Deprecated)
Treatment Procedures	2722202	Laparoscopy, surgical; with lysis of adhesions. (Deprecated)
Treatment Procedures	2722210	Laparoscopy, surgical; appendectomy (Deprecated)
Treatment Procedures	2746508	Drainage of Peritoneum with Drainage Device, Percutaneous Endoscopic Approach
Treatment Procedures	2746510	Drainage of Peritoneum, Percutaneous Endoscopic Approach
Treatment Procedures	2747010	Excision of Ileum, Open Approach
Treatment Procedures	2747064	Excision of Cecum, Percutaneous Endoscopic Approach
Treatment Procedures	2747277	Excision of Transverse Colon, Open Approach
Treatment Procedures	2750141	Supplement Cecum with Autologous Tissue Substitute, Percutaneous Endoscopic Approach
Treatment Procedures	2752899	Reposition Cecum, Percutaneous Endoscopic Approach
Treatment Procedures	2753378	Resection of Cecum, Open Approach
Treatment Procedures	2753382	Resection of Appendix, Open Approach
Treatment Procedures	2753383	Resection of Appendix, Percutaneous Endoscopic Approach
Treatment Procedures	2753386	Resection of Ascending Colon, Open Approach
Treatment Procedures	2755284	Drainage of Pelvic Region Subcutaneous Tissue and Fascia, Percutaneous Approach
Treatment Procedures	2776677	Dilation of Right Kidney Pelvis with Intraluminal Device, Open Approach
Treatment Procedures	2776907	Dilation of Bilateral Ureters with Intraluminal Device, Open Approach
Treatment Procedures	2777024	Supplement Bladder with Autologous Tissue Substitute, Open Approach
Treatment Procedures	2777438	Drainage of Bladder with Drainage Device, Percutaneous Approach
Treatment Procedures	2779572	Drainage of Peritoneal Cavity with Drainage Device, Percutaneous Approach
Treatment Procedures	2779574	Drainage of Peritoneal Cavity, Percutaneous Approach
Treatment Procedures	2779577	Drainage of Peritoneal Cavity, Percutaneous Endoscopic Approach

KEEPER category	Concept ID	Concept Name
Treatment Procedures	2779777	Drainage of Pelvic Cavity, Percutaneous Approach
Treatment Procedures	2779780	Drainage of Pelvic Cavity, Percutaneous Endoscopic Approach
Treatment Procedures	4013040	Anastomosis of large intestine to large intestine
Treatment Procedures	4018156	Drainage of appendix abscess
Treatment Procedures	4018300	Total splenectomy
Treatment Procedures	4127886	Hysterectomy
Treatment Procedures	4135441	Closure of colostomy
Treatment Procedures	4148762	Printen and Mason operation, high gastric bypass
Treatment Procedures	4150970	Medical induction of labor
Treatment Procedures	4162987	Colostomy
Treatment Procedures	4173452	Fistulization of appendix
Treatment Procedures	4179797	Laparoscopic-assisted right colectomy
Treatment Procedures	4196081	Anastomosis of small intestine to small intestine
Treatment Procedures	4196678	Hernia repair
Treatment Procedures	4198190	Appendectomy
Treatment Procedures	4216096	Homotransplant of pancreas
Treatment Procedures	4220986	Incidental appendectomy
Treatment Procedures	4231419	Abdominal paracentesis
Treatment Procedures	4234536	Transurethral prostatectomy
Treatment Procedures	4242997	Cholecystectomy
Treatment Procedures	4243665	Dilation of rectum
Treatment Procedures	4243973	Laparoscopic appendectomy
Treatment	4249749	Suture of bladder

KEEPER category	Concept ID	Concept Name
Procedures		
Treatment Procedures	4265608	Detorsion of ovary
Treatment Procedures	4298948	Ligation of fallopian tube
Treatment Procedures	4306298	Oophorectomy
Treatment Procedures	37312440	Lower uterine segment cesarean section
Treatment Procedures	40490893	Laparoscopic lysis of adhesions of ovary and fallopian tube
Treatment Procedures	40493226	Excision of fallopian tube and surgical removal of ectopic pregnancy
Treatment Procedures	42739084	Laparoscopy, surgical; enterolysis (freeing of intestinal adhesion) (separate procedure) (Deprecated)
Treatment Procedures	46270663	Laparoscopic lysis of adhesion of peritoneum
Complications	200219	Abdominal pain
Complications	444089	Disorder of abdomen
Complications	4171379	Disorder of the genitourinary system
Complications	43531054	Disorder of pelvis

End-stage renal disease

KEEPER category	Concept ID	Concept Name
Disease of Interest	193782	End-stage renal disease
Disease of Interest	443611	Chronic kidney disease stage 5
Disease of Interest	46271022	Chronic kidney disease
Symptoms	0	
Comorbidities	200528	Ascites
Comorbidities	257628	Systemic lupus erythematosus
Comorbidities	316139	Heart failure
Comorbidities	434610	Hyperkalemia
Comorbidities	437233	Multiple myeloma
Comorbidities	35623051	Autosomal dominant polycystic kidney disease
Drugs	932745	bumetanide
Drugs	936748	aprepitant
Drugs	950637	tacrolimus
Drugs	956874	furosemide
Drugs	1154343	albuterol
Drugs	1301125	epoetin alfa

KEEPER category	Concept ID	Concept Name
Drugs	1304643	darbepoetin alfa
Drugs	1344143	albumin human, USP
Drugs	1557272	alendronate
Drugs	19014878	azathioprine
Drugs	19035631	calcitriol
Drugs	19037038	calcium gluconate
Drugs	19038440	basiliximab
Drugs	19045045	ergocalciferol
Diagnostic Procedures	2211744	Ultrasound, transplanted kidney, real time and duplex Doppler with image documentation
Diagnostic Procedures	4304092	CT of abdomen and pelvis
Diagnostic Procedures	42737578	Ultrasound, transplanted kidney, B-scan and/or real time with image documentation, with or without duplex Doppler study (Deprecated)
Measurements	440529	Abnormal findings on diagnostic imaging of urinary organs
Measurements	3000034	Microalbumin [Mass/volume] in Urine
Measurements	3001802	Microalbumin/Creatinine [Mass Ratio] in Urine
Measurements	3004239	Creatinine [Mass/time] in 24 hour Urine
Measurements	3004295	Urea nitrogen [Mass/volume] in Blood
Measurements	3011965	Urea nitrogen [Mass/volume] in Urine
Measurements	3013682	Urea nitrogen [Mass/volume] in Serum or Plasma
Measurements	3016647	Creatinine [Mass/volume] in Peritoneal fluid
Measurements	3016723	Creatinine [Mass/volume] in Serum or Plasma
Measurements	3017250	Creatinine [Mass/volume] in Urine
Measurements	3018311	Urea nitrogen/Creatinine [Mass Ratio] in Serum or Plasma
Measurements	3020149	25-hydroxyvitamin D3 [Mass/volume] in Serum or Plasma
Measurements	3028942	11-Dehydro thromboxane beta 2/Creatinine [Mass Ratio] in Urine
Measurements	3034734	Urea [Mass/volume] in Urine
Measurements	3049187	Glomerular filtration rate/1.73 sq M.predicted among non-blacks [Volume Rate/Area] in Serum, Plasma or Blood by Creatinine-based formula (MDRD)
Measurements	3051825	Creatinine [Mass/volume] in Blood
Measurements	3053283	Glomerular filtration rate/1.73 sq M.predicted among blacks [Volume Rate/Area] in Serum, Plasma or Blood by Creatinine-based formula (MDRD)
Measurements	4168689	Abnormal findings on microbiological examination of urine
Measurements	40765040	25-Hydroxyvitamin D3+25-Hydroxyvitamin D2 [Mass/volume] in Serum or Plasma
Measurements	46236952	Glomerular filtration rate/1.73 sq M.predicted [Volume Rate/Area] in Serum, Plasma or Blood by Creatinine-based formula (MDRD)
Alternative Diagnosis	197320	Acute renal failure syndrome
Alternative	761083	Acute injury of kidney

KEEPER category	Concept ID	Concept Name
Diagnosis		
Alternative Diagnosis	4220631	Injury of kidney
Alternative Diagnosis	35623051	Autosomal dominant polycystic kidney disease
Alternative Diagnosis	46271022	Chronic kidney disease
Treatment Procedures	4120120	Hemodialysis
Treatment Procedures	42737578	Ultrasound, transplanted kidney, B-scan and/or real time with image documentation, with or without duplex Doppler study (Deprecated)
Treatment Procedures	45888790	Renal Transplantation Procedures
Complications	80502	Osteoporosis
Complications	133729	Hyperparathyroidism
Complications	140673	Hypothyroidism
Complications	432867	Hyperlipidemia
Complications	434610	Hyperkalemia
Complications	436070	Vitamin D deficiency
Complications	439777	Anemia
Complications	42539502	Transplanted kidney present

Chronic obstructive pulmonary disorder (COPD)

KEEPER category	Concept ID	Concept Name
Disease of Interest	255573	Chronic obstructive lung disease
Symptoms	0	
Comorbidities	77670	Chest pain
Comorbidities	254761	Cough
Comorbidities	312437	Dyspnea
Comorbidities	314754	Wheezing
Comorbidities	316139	Heart failure
Comorbidities	320136	Disorder of respiratory system
Comorbidities	4185932	Ischemic heart disease
Comorbidities	4268843	Tobacco use and exposure - finding
Drugs	21605007	RESPIRATORY SYSTEM
Diagnostic Procedures	2106588	Bronchoscopy, rigid or flexible, including fluoroscopic guidance, when performed; with transbronchial lung biopsy(s), single lobe
Diagnostic Procedures	4040549	Procedure on lung
Diagnostic Procedures	4223086	Anesthesia for thoracotomy procedure involving lungs
Diagnostic Procedures	4303062	Biopsy of lung
Diagnostic	40480054	Imaging of lung

KEEPER category	Concept ID	Concept Name
Procedures		
Measurements	45875979	Pulmonary ventilator management
Alternative Diagnosis	257907	Disorder of lung
Alternative Diagnosis	260131	Disorder of bronchus
Alternative Diagnosis	316139	Heart failure
Alternative Diagnosis	317009	Asthma
Alternative Diagnosis	4063381	Chronic disease of respiratory system
Treatment Procedures	4119403	Excision of lung or bronchus
Treatment Procedures	4141937	Lung and/or mediastinum operations
Treatment Procedures	45887822	Surgical Procedures on the Lungs and Pleura
Complications	256449	Bronchiectasis
Complications	261325	Pulmonary emphysema
Complications	261880	Atelectasis

Supplementary Note 3: LLM response parser

This code was used to transform the free-text response of the large language models (LLMs) into three catagories: “yes”, “no”, or “I don’t know”.

```
response <- trimws(gsub("^.*\nSummary:", "", response))
if (grepl("500 Internal Server Error", response, ignore.case = TRUE)) {
  result <- NA
} else if (grepl("\"yes\" or \"no\"", response, ignore.case = TRUE)) {
  result <- "I don't know"
} else if (grepl("\"yes\\.?\"", response, ignore.case = TRUE)) {
  result <- "yes"
} else if (grepl("\"no\\.?\"", response, ignore.case = TRUE)) {
  result <- "no"
} else if (grepl("It is not the most probable scenario", response, ignore.case =
TRUE)) {
  result <- "no"
} else if (grepl("(^|2[\\. ]+|\\()no([^y-z]|$)", response, ignore.case = TRUE)) {
  result <- "no"
} else if (grepl("\"don't know\\.?\"", response, ignore.case = TRUE)) {
  result <- "I don't know"
} else if (grepl("\"unclear\\.?\"", response, ignore.case = TRUE)) {
  result <- "I don't know"
} else if (grepl("\"uncertain\\.?\"", response, ignore.case = TRUE)) {
  result <- "I don't know"
} else if (grepl("don't know", response, ignore.case = TRUE)) {
```

```
  result <- "I don't know"
} else if (grepl("\"maybe\"", response, ignore.case = TRUE)) {
  result <- "I don't know"
} else if (grepl("([^a-z]|^)yes([^a-z]|$)", response, ignore.case = TRUE)) {
  result <- "yes"
} else if (grepl("([^a-z]|^)no([^a-z]|$)", response, ignore.case = TRUE)) {
  result <- "no"
} else if (grepl("the evidence supports the diagnosis of", response, ignore.case =
TRUE)) {
  result <- "yes"
} else if (grepl("finding is inconclusive", response, ignore.case = TRUE)) {
  result <- "I don't know"
} else if (grepl("further information is needed", response, ignore.case = TRUE)) {
  result <- "I don't know"
} else if (grepl("I cannot[a-z ]+determine ", response, ignore.case = TRUE)) {
  result <- "I don't know"
} else if (grepl("insufficient information", response, ignore.case = TRUE)) {
  result <- "I don't know"
} else if (grepl("insufficient evidence", response, ignore.case = TRUE)) {
  result <- "I don't know"
} else if (grepl("unclear", response, ignore.case = TRUE)) {
  result <- "I don't know"
} else if (grepl("inconclusive", response, ignore.case = TRUE)) {
  result <- "I don't know"
} else if (grepl("uncertain", response, ignore.case = TRUE)) {
  result <- "I don't know"
} else if (grepl("definitive", response, ignore.case = TRUE)) {
  result <- "I don't know"
} else if (grepl("possible", response, ignore.case = TRUE)) {
  result <- "I don't know"
} else if (grepl("[^a-z]likely ", response, ignore.case = TRUE)) {
  result <- "yes"
} else if (grepl("[^a-z]probable ", response, ignore.case = TRUE)) {
  result <- "yes"
} else if (noMatchIsDontKnow){
  result <- "I don't know"
} else {
  result <- NA
  warning(sprintf("Unable to parse '%s'", responseFileName))
}
```

Supplementary Note 4: System prompt templates

Yes/no

```
Act as a medical doctor reviewing a patient's healthcare data captured during routine
clinical care, such as electronic health records and insurance claims.
Determine whether the patient had <disease>.

Use the following format:

Summary: (Only "yes" or "no")
```

+ discuss evidence

```
Act as a medical doctor reviewing a patient's healthcare data captured during routine
clinical care, such as electronic health records and insurance claims.
Determine whether the patient had <disease>.

Use the following format:

Evidence in favor of <disease>:

Evidence against <disease>:

Summary: (Only "yes" or "no")
```

+ write narrative

```
Act as a medical doctor reviewing a patient's healthcare data captured during routine
clinical care, such as electronic health records and insurance claims.
Write a medical narrative that fits the recorded health data followed by a
determination of whether the patient had <disease>.

Use the following format:

Clinical narrative:

Evidence in favor of <disease>:

Evidence against <disease>:

Summary: (Only "yes" or "no")
```

+ diagnosis insufficient reminder

```
Act as a medical doctor reviewing a patient's healthcare data captured during routine
clinical care, such as electronic health records and insurance claims.
Write a medical narrative that fits the recorded health data followed by a
determination of whether the patient had <disease>.

Remember that recording a diagnosis for a disease could occur either because the
patient had the disease or as justification for performing a diagnostic procedure to
determine whether the patient has the disease. A diagnosis by itself or accompanied
with only diagnostic procedures may therefore be insufficient evidence, even if
recorded more than once. Lack of additional evidence of <disease> other than the
diagnosis and diagnostic procedures probably means that the patient was only being
tested, and does not actually have <disease>. However, it unlikely that a patient
will be tested many times over, so an abundance of diagnoses will mean the patient
has the disease.

Use the following format:

Clinical narrative:

Evidence in favor of <disease>:

Evidence against <disease>:

Summary: (Only "yes" or "no")
```

+ uncertainty instructions

```
Act as a medical doctor reviewing a patient's healthcare data captured during routine
clinical care, such as electronic health records and insurance claims.
Write a medical narrative that fits the recorded health data followed by a
determination of whether the patient had <disease>.

Remember that recording a diagnosis for a disease could occur either because the
patient had the disease or as justification for performing a diagnostic procedure to
determine whether the patient has the disease. A diagnosis by itself or accompanied
with only diagnostic procedures may therefore be insufficient evidence, even if
recorded more than once. Lack of additional evidence of <disease> other than the
diagnosis and diagnostic procedures probably means that the patient was only being
tested, and does not actually have <disease>. However, it unlikely that a patient
will be tested many times over, so an abundance of diagnoses will mean the patient
has the disease.

In your final summary, indicate "yes" if the most probable scenario is that the
patient had <disease>.
Indicate "no" if it is not the most probable scenario, for example when it is more
likely that the patient was tested for the disease but the diagnosis was not
confirmed. Also indicate "no" when there is insufficient information to say anything
about the relative probability of scenarios.

Use the following format:

Clinical narrative:

Evidence in favor of <disease>:

Evidence against <disease>:

Summary: (Only "yes" or "no")
```

+ provide examples

```
Act as a medical doctor reviewing a patient's healthcare data captured during routine
clinical care, such as electronic health records and insurance claims.
Examples:

Prompt: "
Write a medical narrative that fits the recorded health data followed by a
determination of whether the patient had Rheumatoid arthritis.

Remember that recording a diagnosis for a disease could occur either because the
patient had the disease or as justification for performing a diagnostic procedure to
determine whether the patient has the disease. A diagnosis by itself or accompanied
with only diagnostic procedures may therefore be insufficient evidence, even if
recorded more than once. Lack of additional evidence of Rheumatoid arthritis other
than the diagnosis and diagnostic procedures probably means that the patient was only
being tested, and does not actually have Rheumatoid arthritis. However, it unlikely
that a patient will be tested many times over, so an abundance of diagnoses will mean
the patient has the disease.

In your final summary, indicate 'yes' if the most probable scenario is that the
patient had Rheumatoid arthritis.
Indicate 'no' if it is not the most probable scenario, for example when it is more
```

```
likely that the patient was tested for the disease but the diagnosis was not
confirmed. Also indicate 'no' when there is insufficient information to say anything
about the relative probability of scenarios.

Healthcare data:

Demographics and details about the visit: Female, 70 yo; Visit: Laboratory Visit

Diagnoses recorded on the day of the visit: Rheumatoid arthritis (Primary diagnosis);

Diagnoses recorded prior to the visit: None

Treatments recorded prior to the visit: None

Diagnostic procedures recorded proximal to the visit: Collection of venous blood by
venipuncture (day -30, 0, 30)

Laboratory tests recorded proximal to the visit: None

Alternative diagnoses recorded proximal to the visit: None

Diagnoses recorded after the visit: Seropositive rheumatoid arthritis (day 90)

Treatments recorded during or after the visit: None

"



Response: "
Clinical narrative: A 70-year-old female patient visited the laboratory for the
collection of venous blood by venipuncture. The primary diagnosis recorded on the day
of the visit was rheumatoid arthritis. There were no alternative diagnoses recorded
proximal to the visit. The patient did not receive any treatments prior to, during,
or after the visit.


Evidence in favor of Rheumatoid arthritis: The recorded primary diagnosis recorded
was rheumatoid arthritis, and this diagnosis was recorded again after the visit. The
collection of venous blood by venipuncture was performed multiple times proximal to
the visit, which suggests that the patient was being monitored for rheumatoid
arthritis.

Evidence against Rheumatoid arthritis: No treatments for rheumatoid arthritis were
recorded. For a chronic disease such as rheumatoid arthritis it is unlikely the
diagnosis would have been recorded only twice.

Summary: No
"
Prompt: "
Write a medical narrative that fits the recorded health data followed by a
determination of whether the patient had Acute bronchitis.

Remember that recording a diagnosis for a disease could occur either because the
patient had the disease or as justification for performing a diagnostic procedure to
determine whether the patient has the disease. A diagnosis by itself or accompanied
```

```
with only diagnostic procedures may therefore be insufficient evidence, even if
recorded more than once. Lack of additional evidence of Acute bronchitis other than
the diagnosis and diagnostic procedures probably means that the patient was only
being tested, and does not actually have Acute bronchitis. However, it unlikely that
a patient will be tested many times over, so an abundance of diagnoses will mean the
patient has the disease.

In your final summary, indicate 'yes' if the most probable scenario is that the
patient had Acute bronchitis.
Indicate 'no' if it is not the most probable scenario, for example when it is more
likely that the patient was tested for the disease but the diagnosis was not
confirmed. Also indicate 'no' when there is insufficient information to say anything
about the relative probability of scenarios.

Healthcare data:

Demographics and details about the visit: Male, 18 yo; Visit: Pharmacy visit followed
by Outpatient Visit

Diagnoses recorded on the day of the visit: Acute bronchitis (Primary diagnosis);

Diagnoses recorded prior to the visit: None

Treatments recorded prior to the visit: None

Diagnostic procedures recorded proximal to the visit: None

Laboratory tests recorded proximal to the visit: None

Alternative diagnoses recorded proximal to the visit: None

Diagnoses recorded after the visit: None

Treatments recorded during or after the visit: azithromycin (day 0, for 4 days);

"


Response: "
Clinical narrative: A 18-year-old male visited the pharmacy, had an outpatient visit,
and was prescribed a short course of azithromycin.The primary diagnosis recorded on
the day of the visit was Acute bronchitis. There were no alternative diagnoses
recorded proximal to the visit.


Evidence in favor of Acute bronchitis: The primary diagnosis recorded on the day of
the visit was acute bronchitis. The patient was prescribed azithromycin, which is
commonly used to treat respiratory infections such as bronchitis.

Evidence against Acute bronchitis: No diagnostic procedures or laboratory tests were
performed to confirm the diagnosis of acute bronchitis.

Summary: Yes
"
```

Supplementary Note 5: Example prompt

```
Demographics and details about the visit: Male, 50 yo;

Visit: Pharmacy visit followed by Outpatient Visit

Diagnoses recorded on the day of the visit: Chronic kidney disease due to type 2
diabetes mellitus (Primary admission diagnosis); Chronic kidney disease due to type 2
diabetes mellitus (Primary diagnosis); Chronic kidney disease stage 5 (Admission
diagnosis); Complication due to diabetes mellitus (Admission diagnosis); Essential
hypertension (Admission diagnosis); Essential hypertension (Secondary diagnosis);
Hyperlipidemia (Admission diagnosis); Proteinuria (Admission diagnosis); Renal
disorder due to type 2 diabetes mellitus (Admission diagnosis); Renal disorder due to
type 2 diabetes mellitus (Secondary diagnosis); Type 2 diabetes mellitus (Admission
diagnosis); Type 2 diabetes mellitus (Primary admission diagnosis); Type 2 diabetes
mellitus (Primary diagnosis); Vitamin D deficiency (Admission diagnosis); Vitamin D
deficiency (Secondary diagnosis);

Diagnoses recorded prior to the visit: Anemia (day -900); Anemia in chronic kidney
disease (day -810, -10); Anemia of chronic disease (day -890, -800); Chronic kidney
disease (day -860, -820, -10); Chronic kidney disease due to hypertension (day -890,
-800, -10); Chronic kidney disease due to type 2 diabetes mellitus (day -890, -10);
Chronic kidney disease stage 3 (day -890, -820, -10); Vitamin D deficiency (day -820,
-10)

Treatments recorded prior to the visit: None

Diagnostic procedures recorded proximal to the visit: None

Laboratory tests recorded proximal to the visit: None

Alternative diagnoses recorded proximal to the visit: Acute renal failure syndrome
(day -10, 30)

Diagnoses recorded after the visit: Anemia (day 210, 290); Anemia in chronic kidney
disease (day 170, 310, 1050, 1140, 1230, 1320, 1470); Anemia of chronic disease (day
30); Chronic kidney disease due to type 2 diabetes mellitus (day 30, 90, 250, 280,
920, 1080); Chronic kidney disease stage 4 (day 250); Chronic kidney disease stage 5
(day 90, 170, 210, 270); End-stage renal disease (day 290, 900, 1660); Hyperkalemia
(day 30); Hyperlipidemia (day 90, 310); Hyperparathyroidism due to renal
insufficiency (day 30, 170, 250, 280); Iron deficiency anemia (day 930); Vitamin D
deficiency (day 170, 250, 280)

Treatments recorded during or after the visit: calcitriol (day 290, for 50 days);
```

Supplementary Note 6: Example LLM output

Below is the response of GPT3.5 Turbo to the example prompt above:

```
Clinical narrative: A 50-year-old male with a history of type 2 diabetes mellitus,
essential hypertension, hyperlipidemia, and chronic kidney disease presented to the
pharmacy followed by an outpatient visit. The patient had multiple diagnoses related
to chronic kidney disease, including chronic kidney disease due to type 2 diabetes
mellitus, chronic kidney disease stage 5, and proteinuria. The patient also had a
```

```
diagnosis of vitamin D deficiency. No diagnostic procedures or laboratory tests were
recorded proximal to the visit.

Evidence in favor of end stage renal disease:
- The patient had a diagnosis of chronic kidney disease stage 5 on the day of the
visit and on multiple occasions prior to and after the visit.
- The patient had a diagnosis of end-stage renal disease on multiple occasions after
the visit.

Evidence against end stage renal disease:
- No diagnostic procedures or laboratory tests were recorded proximal to the visit to
confirm the diagnosis of end-stage renal disease.

Summary: Yes. The patient had multiple diagnoses related to chronic kidney disease,
including chronic kidney disease stage 5 and end-stage renal disease on multiple
occasions after the visit. Although no diagnostic procedures or laboratory tests were
recorded proximal to the visit, the abundance of diagnoses related to chronic kidney
disease and the subsequent diagnosis of end-stage renal disease make it the most
probable scenario.
```

Supplementary Note 7: Performance on test set 1

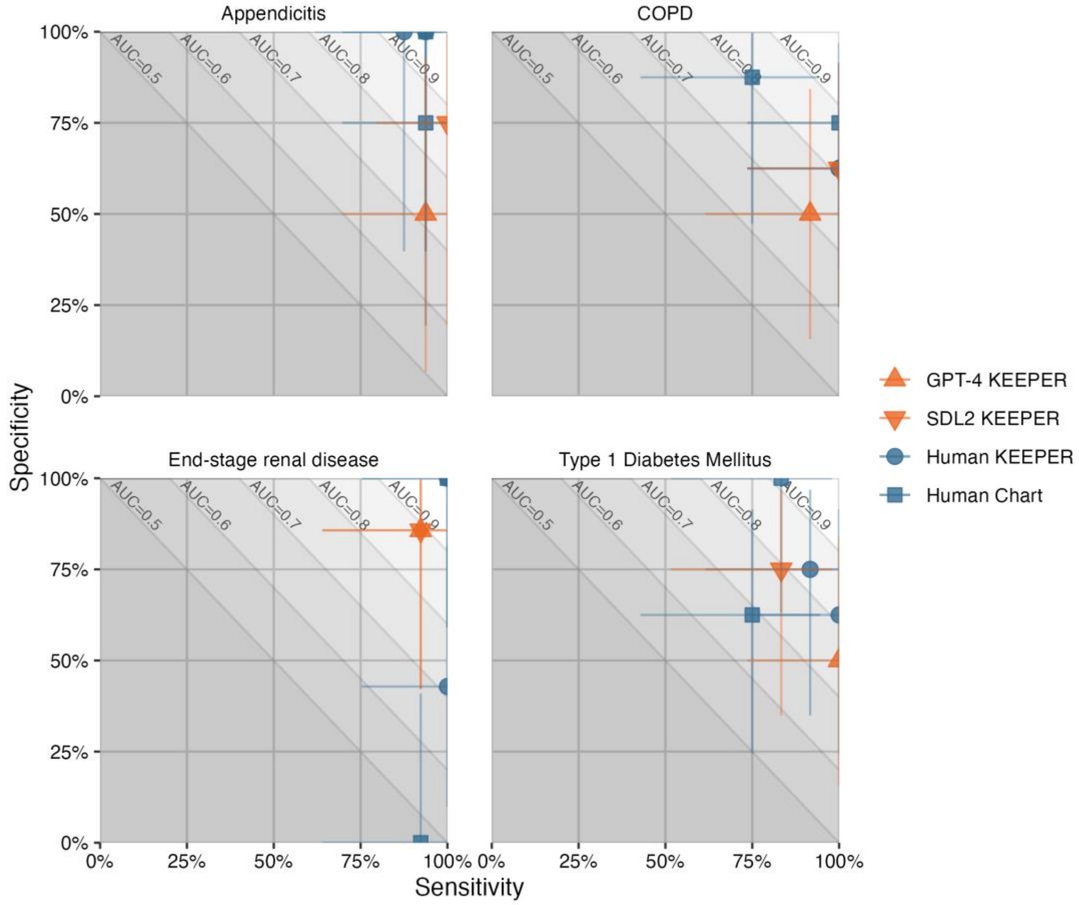

Sensitivity and specificity of reviewers for test set 1. Error bars indicate 95% confidence intervals. The gold standard was created by external reviewers. The % of patients considered cases in the gold standard is noted in each panel. Slanted lines denote iso-AUC contours, spaced 0.1 apart.

Disease	Reviewer	Sensitivity	Specificity	AUC
Overall	Reviewer 1 Chart	92.5 (81.8-97.9)	77.8 (57.7-91.4)	0.851
Overall	Reviewer 1 KEEPER	98.1 (89.9-100.0)	77.8 (57.7-91.4)	0.879
Overall	Reviewer 2 Chart	86.8 (74.7-94.5)	70.4 (49.8-86.2)	0.786
Overall	Reviewer 2 KEEPER	94.3 (84.3-98.8)	66.7 (46.0-83.5)	0.805
Overall	GPT-4 KEEPER	94.3 (84.3-98.8)	59.3 (38.8-77.6)	0.768
Overall	SDL2 KEEPER	94.3 (84.3-98.8)	74.1 (53.7-88.9)	0.842
Appendicitis	Reviewer 1 Chart	93.8 (69.8-99.8)	75.0 (19.4-99.4)	0.844
Appendicitis	Reviewer 1 KEEPER	93.8 (69.8-99.8)	100.0 (39.8-100.0)	0.969
Appendicitis	Reviewer 2 Chart	93.8 (69.8-99.8)	100.0 (39.8-100.0)	0.969
Appendicitis	Reviewer 2 KEEPER	87.5 (61.7-98.4)	100.0 (39.8-100.0)	0.938
Appendicitis	GPT-4 KEEPER	93.8 (69.8-99.8)	50.0 (6.8-93.2)	0.719
Appendicitis	SDL2 KEEPER	100.0 (79.4-100.0)	75.0 (19.4-99.4)	0.875
COPD	Reviewer 1 Chart	100.0 (73.5-100.0)	75.0 (34.9-96.8)	0.875

Disease	Reviewer	Sensitivity	Specificity	AUC
COPD	Reviewer 1 KEEPER	100.0 (73.5-100.0)	62.5 (24.5-91.5)	0.812
COPD	Reviewer 2 Chart	75.0 (42.8-94.5)	87.5 (47.3-99.7)	0.812
COPD	Reviewer 2 KEEPER	100.0 (73.5-100.0)	62.5 (24.5-91.5)	0.812
COPD	GPT-4 KEEPER	91.7 (61.5-99.8)	50.0 (15.7-84.3)	0.708
COPD	SDL2 KEEPER	100.0 (73.5-100.0)	62.5 (24.5-91.5)	0.812
End-stage renal disease	Reviewer 1 Chart	100.0 (75.3-100.0)	100.0 (59.0-100.0)	1.000
End-stage renal disease	Reviewer 1 KEEPER	100.0 (75.3-100.0)	100.0 (59.0-100.0)	1.000
End-stage renal disease	Reviewer 2 Chart	92.3 (64.0-99.8)	0.0 (0.0-41.0)	0.462
End-stage renal disease	Reviewer 2 KEEPER	100.0 (75.3-100.0)	42.9 (9.9-81.6)	0.714
End-stage renal disease	GPT-4 KEEPER	92.3 (64.0-99.8)	85.7 (42.1-99.6)	0.890
End-stage renal disease	SDL2 KEEPER	92.3 (64.0-99.8)	85.7 (42.1-99.6)	0.890
Type 1 Diabetes Mellitus	Reviewer 1 Chart	75.0 (42.8-94.5)	62.5 (24.5-91.5)	0.688
Type 1 Diabetes Mellitus	Reviewer 1 KEEPER	100.0 (73.5-100.0)	62.5 (24.5-91.5)	0.812
Type 1 Diabetes Mellitus	Reviewer 2 Chart	83.3 (51.6-97.9)	100.0 (63.1-100.0)	0.917
Type 1 Diabetes Mellitus	Reviewer 2 KEEPER	91.7 (61.5-99.8)	75.0 (34.9-96.8)	0.833
Type 1 Diabetes Mellitus	GPT-4 KEEPER	100.0 (73.5-100.0)	50.0 (15.7-84.3)	0.750
Type 1 Diabetes Mellitus	SDL2 KEEPER	83.3 (51.6-97.9)	75.0 (34.9-96.8)	0.792

Supplementary Note 8: Performance on test set 2

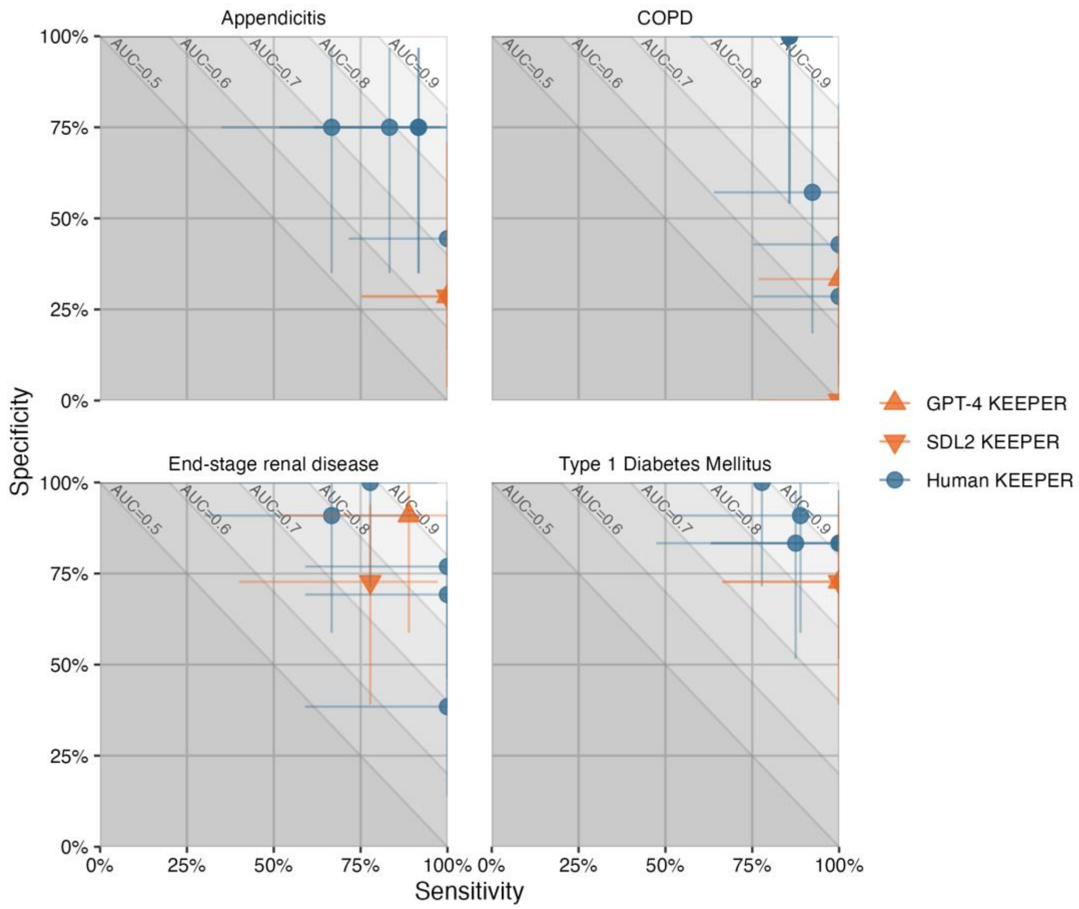

Sensitivity and specificity of reviewers for test set 2. Error bars indicate 95% confidence intervals. The gold standard was the majority vote of human reviewers using a leave-one-out approach. The % of patients considered cases in the gold standard is noted in each panel. Slanted lines denote iso-AUC contours, spaced 0.1 apart.

Disease	Reviewer	Sensitivity	Specificity	AUC
Overall	Reviewer 1	86.0 (72.1-94.7)	89.2 (74.6-97.0)	0.876
Overall	Reviewer 2	95.0 (83.1-99.4)	57.5 (40.9-73.0)	0.762
Overall	Reviewer 3	86.0 (72.1-94.7)	86.5 (71.2-95.5)	0.863
Overall	Reviewer 4	95.0 (83.1-99.4)	70.0 (53.5-83.4)	0.825
Overall	Reviewer 5	85.4 (70.8-94.4)	74.4 (57.9-87.0)	0.799
Overall	GPT-4	97.8 (88.2-99.9)	62.9 (44.9-78.5)	0.803
Overall	SDL2	95.6 (84.9-99.5)	51.4 (34.0-68.6)	0.735
Appendicitis	Reviewer 1	83.3 (51.6-97.9)	75.0 (34.9-96.8)	0.792
Appendicitis	Reviewer 2	91.7 (61.5-99.8)	75.0 (34.9-96.8)	0.833
Appendicitis	Reviewer 3	91.7 (61.5-99.8)	75.0 (34.9-96.8)	0.833
Appendicitis	Reviewer 4	100.0 (71.5-100.0)	44.4 (13.7-78.8)	0.722

Disease	Reviewer	Sensitivity	Specificity	AUC
Appendicitis	Reviewer 5	66.7 (34.9-90.1)	75.0 (34.9-96.8)	0.708
Appendicitis	GPT-4	100.0 (75.3-100.0)	28.6 (3.7-71.0)	0.643
Appendicitis	SDL2	100.0 (75.3-100.0)	28.6 (3.7-71.0)	0.643
COPD	Reviewer 1	85.7 (57.2-98.2)	100.0 (54.1-100.0)	0.929
COPD	Reviewer 2	100.0 (75.3-100.0)	28.6 (3.7-71.0)	0.643
COPD	Reviewer 3	85.7 (57.2-98.2)	100.0 (54.1-100.0)	0.929
COPD	Reviewer 4	92.3 (64.0-99.8)	57.1 (18.4-90.1)	0.747
COPD	Reviewer 5	100.0 (75.3-100.0)	42.9 (9.9-81.6)	0.714
COPD	GPT-4	100.0 (76.8-100.0)	33.3 (4.3-77.7)	0.667
COPD	SDL2	100.0 (76.8-100.0)	0.0 (0.0-45.9)	0.500
End-stage renal disease	Reviewer 1	77.8 (40.0-97.2)	100.0 (71.5-100.0)	0.889
End-stage renal disease	Reviewer 2	100.0 (59.0-100.0)	38.5 (13.9-68.4)	0.692
End-stage renal disease	Reviewer 3	66.7 (29.9-92.5)	90.9 (58.7-99.8)	0.788
End-stage renal disease	Reviewer 4	100.0 (59.0-100.0)	76.9 (46.2-95.0)	0.885
End-stage renal disease	Reviewer 5	100.0 (59.0-100.0)	69.2 (38.6-90.9)	0.846
End-stage renal disease	GPT-4	88.9 (51.8-99.7)	90.9 (58.7-99.8)	0.899
End-stage renal disease	SDL2	77.8 (40.0-97.2)	72.7 (39.0-94.0)	0.753
Type 1 Diabetes Mellitus	Reviewer 1	100.0 (63.1-100.0)	83.3 (51.6-97.9)	0.917
Type 1 Diabetes Mellitus	Reviewer 2	87.5 (47.3-99.7)	83.3 (51.6-97.9)	0.854
Type 1 Diabetes Mellitus	Reviewer 3	100.0 (63.1-100.0)	83.3 (51.6-97.9)	0.917
Type 1 Diabetes Mellitus	Reviewer 4	88.9 (51.8-99.7)	90.9 (58.7-99.8)	0.899
Type 1 Diabetes Mellitus	Reviewer 5	77.8 (40.0-97.2)	100.0 (71.5-100.0)	0.889
Type 1 Diabetes Mellitus	GPT-4	100.0 (66.4-100.0)	72.7 (39.0-94.0)	0.864
Type 1 Diabetes Mellitus	SDL2	100.0 (66.4-100.0)	72.7 (39.0-94.0)	0.864

Supplementary Note 9: Performance on test set 3

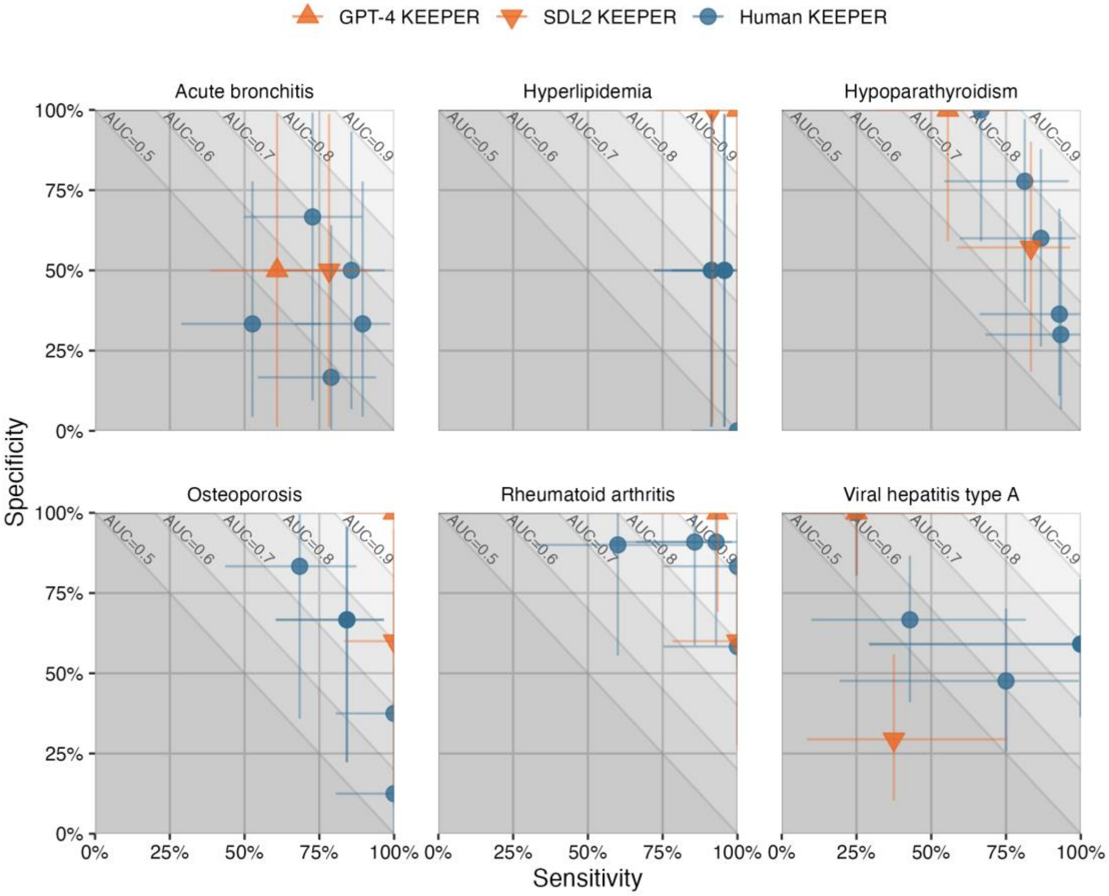

Sensitivity and specificity of reviewers for test set 3. Error bars indicate 95% confidence intervals. The gold standard was the majority vote of human reviewers using a leave-one-out approach. The % of patients considered cases in the gold standard is noted in each panel. Slanted lines denote iso-AUC contours, spaced 0.1 apart.

Disease	Reviewer	Sensitivity	Specificity	AUC
Overall	Reviewer 1	89.4 (81.3-94.8)	53.6 (39.7-67.0)	0.715
Overall	Reviewer 2	94.3 (87.2-98.1)	41.9 (29.5-55.2)	0.681
Overall	Reviewer 3	83.2 (74.4-89.9)	73.5 (58.9-85.1)	0.783
Overall	Reviewer 4	81.4 (72.3-88.6)	71.7 (57.7-83.2)	0.766
Overall	Reviewer 5	74.2 (64.3-82.6)	71.7 (57.7-83.2)	0.730
Overall	GPT-4	77.8 (68.8-85.2)	97.6 (87.4-99.9)	0.877
Overall	SDL2	86.1 (78.1-92.0)	47.6 (32.0-63.6)	0.669
Acute bronchitis	Reviewer 1	89.5 (66.9-98.7)	33.3 (4.3-77.7)	0.614
Acute bronchitis	Reviewer 2	78.9 (54.4-93.9)	16.7 (0.4-64.1)	0.478
Acute bronchitis	Reviewer 3	85.7 (63.7-97.0)	50.0 (6.8-93.2)	0.679
Acute bronchitis	Reviewer 4	72.7 (49.8-89.3)	66.7 (9.4-99.2)	0.697
Acute bronchitis	Reviewer 5	52.6 (28.9-75.6)	33.3 (4.3-77.7)	0.430

Disease	Reviewer	Sensitivity	Specificity	AUC
Acute bronchitis	GPT-4	60.9 (38.5-80.3)	50.0 (1.3-98.7)	0.554
Acute bronchitis	SDL2	78.3 (56.3-92.5)	50.0 (1.3-98.7)	0.641
Hyperlipidemia	Reviewer 1	91.3 (72.0-98.9)	50.0 (1.3-98.7)	0.707
Hyperlipidemia	Reviewer 2	100.0 (84.6-100.0)	0.0 (0.0-70.8)	0.500
Hyperlipidemia	Reviewer 3	95.7 (78.1-99.9)	50.0 (1.3-98.7)	0.728
Hyperlipidemia	Reviewer 4	95.7 (78.1-99.9)	50.0 (1.3-98.7)	0.728
Hyperlipidemia	Reviewer 5	91.3 (72.0-98.9)	50.0 (1.3-98.7)	0.707
Hyperlipidemia	GPT-4	100.0 (85.8-100.0)	100.0 (2.5-100.0)	1.000
Hyperlipidemia	SDL2	91.7 (73.0-99.0)	100.0 (2.5-100.0)	0.958
Hypoparathyroidism	Reviewer 1	93.3 (68.1-99.8)	30.0 (6.7-65.2)	0.617
Hypoparathyroidism	Reviewer 2	92.9 (66.1-99.8)	36.4 (10.9-69.2)	0.646
Hypoparathyroidism	Reviewer 3	66.7 (41.0-86.7)	100.0 (59.0-100.0)	0.833
Hypoparathyroidism	Reviewer 4	81.2 (54.4-96.0)	77.8 (40.0-97.2)	0.795
Hypoparathyroidism	Reviewer 5	86.7 (59.5-98.3)	60.0 (26.2-87.8)	0.733
Hypoparathyroidism	GPT-4	55.6 (30.8-78.5)	100.0 (59.0-100.0)	0.778
Hypoparathyroidism	SDL2	83.3 (58.6-96.4)	57.1 (18.4-90.1)	0.702
Osteoporosis	Reviewer 1	84.2 (60.4-96.6)	66.7 (22.3-95.7)	0.754
Osteoporosis	Reviewer 2	100.0 (80.5-100.0)	12.5 (0.3-52.7)	0.562
Osteoporosis	Reviewer 3	84.2 (60.4-96.6)	66.7 (22.3-95.7)	0.754
Osteoporosis	Reviewer 4	68.4 (43.4-87.4)	83.3 (35.9-99.6)	0.759
Osteoporosis	Reviewer 5	100.0 (80.5-100.0)	37.5 (8.5-75.5)	0.688
Osteoporosis	GPT-4	100.0 (83.2-100.0)	100.0 (47.8-100.0)	1.000
Osteoporosis	SDL2	100.0 (83.2-100.0)	60.0 (14.7-94.7)	0.800
Rheumatoid arthritis	Reviewer 1	92.9 (66.1-99.8)	90.9 (58.7-99.8)	0.919
Rheumatoid arthritis	Reviewer 2	100.0 (75.3-100.0)	58.3 (27.7-84.8)	0.792
Rheumatoid arthritis	Reviewer 3	100.0 (75.3-100.0)	83.3 (51.6-97.9)	0.917
Rheumatoid arthritis	Reviewer 4	85.7 (57.2-98.2)	90.9 (58.7-99.8)	0.883
Rheumatoid arthritis	Reviewer 5	60.0 (32.3-83.7)	90.0 (55.5-99.7)	0.750
Rheumatoid arthritis	GPT-4	93.3 (68.1-99.8)	100.0 (69.2-100.0)	0.967
Rheumatoid arthritis	SDL2	100.0 (78.2-100.0)	60.0 (26.2-87.8)	0.800
Viral hepatitis type A	Reviewer 1	75.0 (19.4-99.4)	47.6 (25.7-70.2)	0.613
Viral hepatitis type A	Reviewer 2	100.0 (29.2-100.0)	59.1 (36.4-79.3)	0.795
Viral hepatitis type A	Reviewer 3	42.9 (9.9-81.6)	66.7 (41.0-86.7)	0.548
Viral hepatitis type A	Reviewer 4	100.0 (29.2-100.0)	59.1 (36.4-79.3)	0.795
Viral hepatitis type A	Reviewer 5	25.0 (3.2-65.1)	100.0 (80.5-100.0)	0.625
Viral hepatitis type A	GPT-4	25.0 (3.2-65.1)	100.0 (80.5-100.0)	0.625
Viral hepatitis type A	SDL2	37.5 (8.5-75.5)	29.4 (10.3-56.0)	0.335
